# Ontogeny of CpG island methylation and specificity of DNMT3 methyltransferases during embryonic development in the mouse

**DOI:** 10.1186/s13059-014-0545-5

**Published:** 2014-12-03

**Authors:** Ghislain Auclair, Sylvain Guibert, Ambre Bender, Michael Weber

**Affiliations:** CNRS, University of Strasbourg, UMR 7242 Biotechnology and Cell Signaling, 300 Bd Sébastien Brant, BP 10413, 67412 Illkirch, France

## Abstract

**Background:**

In the mouse, the patterns of DNA methylation are established during early embryonic development in the epiblast. We quantified the targets and kinetics of DNA methylation acquisition in epiblast cells, and determined the contribution of the *de novo* methyltransferases DNMT3A and DNMT3B to this process.

**Results:**

We generated single-base maps of DNA methylation from the blastocyst to post-implantation stages and in embryos lacking DNMT3A or DNMT3B activity. DNA methylation is established within two days of implantation between embryonic days 4.5 and 6.5. The kinetics of *de novo* methylation are uniform throughout the genome, suggesting a random mechanism of deposition. In contrast, many CpG islands acquire methylation slowly in late epiblast cells. Five percent of CpG islands gain methylation and are found in the promoters of germline genes and in exons of important developmental genes. The onset of global methylation correlates with the upregulation of *Dnmt3a/b* genes in the early epiblast. DNMT3A and DNMT3B act redundantly to methylate the bulk genome and repetitive elements, whereas DNMT3B has a prominent role in the methylation of CpG islands on autosomes and the X chromosome. Reduced CpG island methylation in *Dnmt3b*-deficient embryos correlates with gene reactivation in promoters but reduced transcript abundance in gene bodies. Finally, DNMT3B establishes secondary methylation marks at imprinted loci, which distinguishes *bona fide* germline from somatic methylation imprints.

**Conclusions:**

We reveal that the DNMT3 *de novo* methyltransferases play both redundant and specific functions in the establishment of DNA methylation in the mouse embryo.

**Electronic supplementary material:**

The online version of this article (doi:10.1186/s13059-014-0545-5) contains supplementary material, which is available to authorized users.

## Background

Methylation of cytosines is an epigenetic mark of DNA with crucial functions in mammalian development and diseases. In mammals, methylation occurs almost exclusively in the context of cytosine-guanine (CpG) dinucleotides, which are found at higher frequency in short regions termed CpG islands (CGIs). Paradoxically, the majority of CGIs remain unmethylated in all cell lineages [1-3]. The contrast between the low methylation at CGIs and the high methylation in CpG-poor sequences arises from the accelerated mutational loss of methylated CpGs over evolutionary time [2].

DNA methylation is reprogrammed during embryonic development. The spermatozoa and oocyte acquire differential methylation at many sequences, which establishes a strong epigenetic asymmetry between the gametes that is not limited to imprinted regions [4-6]. After fertilization, these gametic profiles are globally erased to reach a low point of methylation at the blastocyst stage [4-8]. Yet numerous sequences maintain partial methylation in the blastocysts, primarily on the oocyte-derived allele [4-8]. In the case of imprinted regions, this differential allelic methylation is stably maintained throughout embryogenesis and adulthood at a small number of germline differentially methylated regions (gDMRs). After implantation of the embryo, DNA methylation is restored to high levels in epiblast cells throughout the genome as well as at a small number of CGIs [6-8].

Cytosine methylation is catalyzed by DNA methyltransferases (DNMTs). DNMT1 copies methylation on the new DNA strand at hemimethylated CpG sites after DNA replication, which mediates epigenetic inheritance in dividing cells. In contrast, DNMT3A and DNMT3B mediate *de novo* methylation by targeting previously unmethylated CpGs. Another member of the family, DNMT3L, lacks enzymatic activity but acts as a cofactor that stimulates the activity of DNMT3A and DNMT3B in germ cells [4,5]. The knockout of DNMT1 and DNMT3B in mice leads to mid-gestation lethality, indicating that DNA methylation is essential for development [9,10]. DNMT3A knockout animals survive until birth but die at around 4 weeks of age [10].

Advances with high-throughput methods led to a better characterization of the distribution of DNA methylation in mouse embryos, yet several points remain to be clarified, such as (i) the timing of acquisition of DNA methylation in embryos, (ii) the identity and role of CGIs that gain methylation, and (iii) the contribution of DNMT3A and DNMT3B to *de novo* methylation in the embryo. So far the single-gene studies have indicated that DNMT3B methylates the promoters of a few germline genes [7,11] and protocadherin genes [12], whereas DNMT3A and DNMT3B cooperate to methylate other sequences in the mouse embryo [10,13,14]. This suggests that DNMT3A and DNMT3B have both specific and overlapping functions in embryonic methylation, which has not been investigated in a systematic way.

To answer these questions, we generated a single base atlas of cytosine methylation by reduced representation bisulfite sequencing (RRBS) throughout mouse embryonic development starting from the blastocyst stage, as well as in *Dnmt3a* and *Dnmt3b* mutant embryos. We show that methylation is established rapidly at the time of implantation by the combined action of DNMT3A and DNMT3B. In contrast, CGIs behave as a functionally distinct class of sequences that acquire methylation slowly mediated primarily by DNMT3B. We provide a comprehensive analysis of the targets of CGI methylation in development and studied their impact on gene expression in embryos. Our study provides insights into the role and target specificities of the DNMT3 enzymes in mouse development.

## Results

### Temporal mapping of DNA methylation acquisition during murine embryogenesis

**Figure 1:**
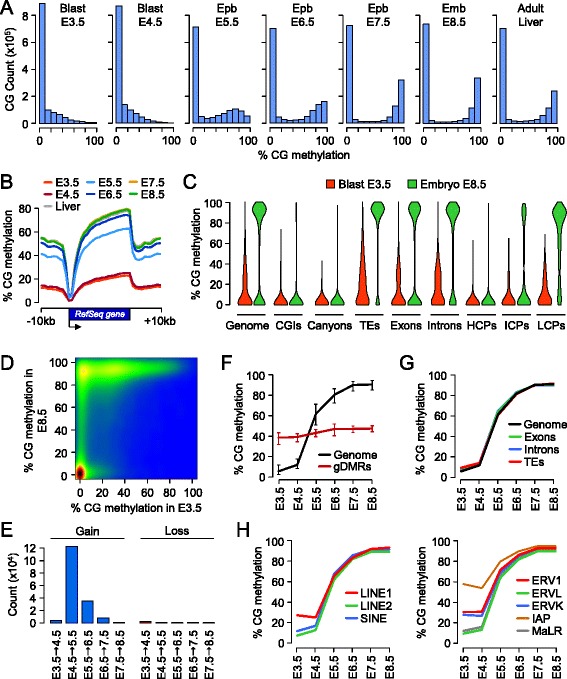
**Acquisition of CpG methylation occurs at implantation in mouse embryos. (A)** Density histograms showing the distribution of methylation levels measured at individual CpGs throughout embryonic development. Blast, blastocyst; Epb, epiblast; Emb, embryo. **(B)** Distribution on CpG methylation in RefSeq genes and 10 kb of flanking sequences throughout embryonic development. For each protein-coding RefSeq gene (excluding the X and Y chromosomes), we calculated methylation in 20 equal-sized windows within the gene and 10 1-kb windows of flanking sequences. **(C)** Violin plots showing the acquisition of CpG methylation in post-implantation embryos compared with blastocysts in various genome elements. TEs, transposable elements; HCPs, high CpG promoters; ICPs, intermediate CpG promoters; LCPs, low CpG promoters. **(D)** Pairwise comparison of CpG methylation (measured in 400 bp tiles) in E3.5 blastocysts and E8.5 post-implantation embryos. The density of points increases from blue to dark red. **(E)** Number of 400 bp tiles that gain or lose more than 20% CpG methylation at each developmental transition. **(F)** Kinetics of *de novo* DNA methylation in development. We selected all the genomic tiles (400 bp) that gain methylation in post-implantation embryos (defined as <20% methylation in E3.5 blastocysts and >50% methylation in E8.5 embryos) and then plotted their methylation as a function of the developmental stage (black line). The red line shows the methylation for 17 imprinted germline DMRs. The lines represent the median methylation and the error bars represent the 25th and 75th percentiles. **(G)** Kinetics of *de novo* DNA methylation in exons, introns and transposable elements (TEs). **(H)** Kinetics of *de novo* DNA methylation in classes of transposable elements. LINE, long interspersed nuclear element; SINE, short interspersed nuclear element. In **(G,H)**, the lines depict the median methylation measured at each developmental stage.

To characterize the wave of *de novo* DNA methylation in the mouse embryo, we generated single-base profiles of cytosine methylation by RRBS at consecutive stages of development between embryonic day (E)3.5 and E8.5 (Figure S1A,B in Additional file 1). We quantified methylation for approximately 1,300,000 CpGs per sample at an average sequencing depth of 68× (Figure S1A in Additional file 1). As shown previously [4-7], the genome of E3.5 blastocysts is globally hypomethylated but contains sequences with partial methylation caused by incomplete erasure of gametic methylation (Figure 1A). After E3.5, cytosine methylation progressively accumulates after implantation exclusively in a CG sequence context (Figure S1C in Additional file 1). During this period, gene bodies, transposable elements and CpG-poor promoters are *de novo* methylated, whereas CpG-rich sequences are protected from methylation (Figure 1B,C). ‘Canyons’, a class of extended regions of low methylation that span loci of developmental transcription factors [15], also form after implantation through protection from *de novo* methylation (Figure 1C). Interestingly, most sequences with partial methylation in blastocysts gain full methylation in post-implantation embryos (Figure 1D); thus, resistance to demethylation after fertilization predisposes to *de novo* methylation in post-implantation embryos. We then investigated the dynamics of methylation by averaging methylation in 400 bp tiles and searching for tiles that gain or lose methylation at each developmental transition. The most dramatic wave of *de novo* methylation occurs in early epiblast between E4.5 and E5.5 (Figure 1E). In contrast there are very few demethylation events at any of the developmental transitions (Figure 1E). This demonstrates that *de novo* methylation occurs rapidly at the time of implantation and is unidirectional. To precisely quantify the kinetics of *de novo* methylation, we selected all tiles that gain methylation in post-implantation embryos and plotted their methylation as a function of the developmental stage (Figure 1F). Methylation increases rapidly from 12% to 62% between E4.5 and E5.5, and reaches almost maximum levels at E6.5 (Figure 1F). As a control, we show that the imprinted gDMRs have stable methylation between 40 and 50% at all stages (Figure 1F). To ask if the rate of *de novo* methylation varies along the genome, we monitored methylation separately in exons, introns and transposable elements and found that they acquire methylation with similar kinetics (Figure 1G). We also monitored methylation in classes of transposable elements and found that, while they have varying degrees of methylation in blastocysts, they all gain methylation with similar kinetics in post-implantation embryos (Figure 1H). This shows that *de novo* methylation occurs rapidly within two days around implantation and is uniform throughout the genome.

### Kinetics of CpG island methylation in developing embryos

**Figure 2:**
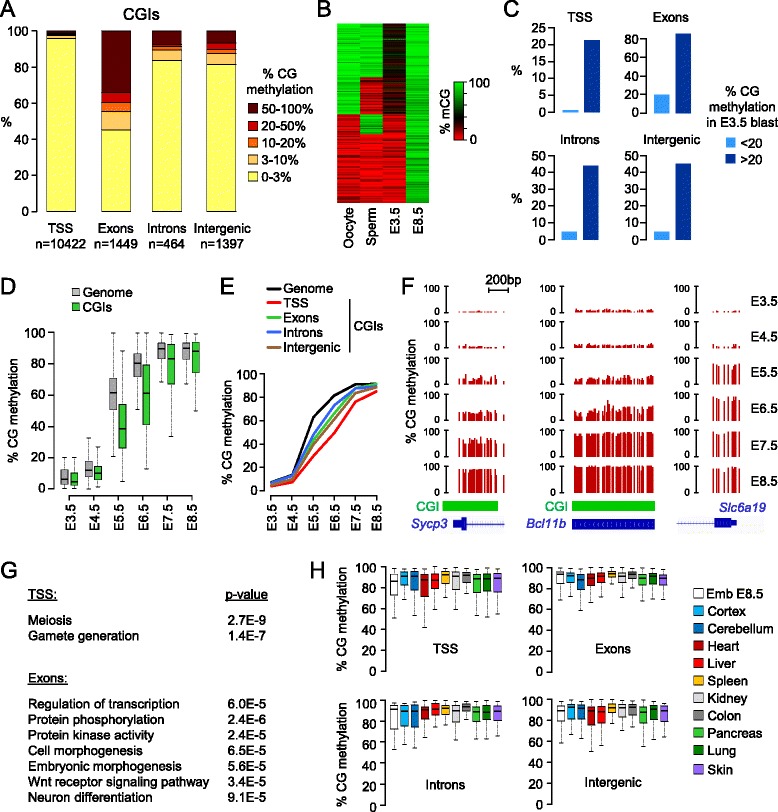
**Targets and kinetics of CpG island methylation in epiblast cells. (A)** Distribution of E8.5 methylation scores in UCSC CGIs located in promoters (-1,000 to 1,000 bp from RefSeq transcription start sites (TSSs)), exons, introns and intergenic sequences. X-linked CGIs are excluded from this analysis. **(B)** Heatmap showing the methylation in gametes and blastocysts for all the CGIs methylated (>50%) in E8.5 embryos. Approximately half of the CGIs inherit partial methylation from the oocyte. **(C)** Percentage of CGIs that gain >50% methylation in E8.5 embryos depending on their methylation in blastocysts. CGIs with oocyte-derived methylation in blastocysts have a much higher probability to gain methylation in post-implantation embryos. **(D)** Kinetics of *de novo* methylation in CGIs compared with the genome (measured in 400 bp genomic tiles). The graph depicts *de novo* methylated sequences defined as <20% methylation in E3.5 blastocysts and >50% methylation in E8.5 embryos. **(E)** Kinetics of *de novo* methylation for CGIs in TSSs, exons, introns and intergenic sequences (selected as <20% methylation in E3.5 blastocysts and >50% methylation in E8.5 embryos) compared with the whole genome (black line). The lines depict the median methylation at each stage. **(F)** Examples of single-CpG RRBS profiles at CGIs with delayed DNA methylation in the promoter of *Sycp3* and one exon of *Blc11b* (chr12:107,915,284-107,917,294). Here and in other figures, the green bars depict the position of the CGI. In comparison, the CpG-poor promoter of the *Slc6a19* gene gains methylation between E4.5 and E5.5. **(G)** Gene Ontology terms associated with methylated CGIs (>50% methylation in E8.5 embryos) in TSSs and exons. **(H)** Distribution of methylation in adult tissues [3] for CGIs with >50% methylation in E8.5 embryos.

Next we focused on CGIs. Out of the 16,023 UCSC CGIs, 89% are covered in each sample (Figure S1D in Additional file 1) and 14,085 have methylation data in all samples. Out of these 14,085 CGIs, 713 (5%) acquire more than 50% methylation in post-implantation embryos (Additional file 2). Remarkably, the proportion of methylated CGIs with more than 50% methylation is much lower at transcription start sites (TSSs; 0.6%, 69/10,694 in total, 63/10,422 on autosomes) than in intergenic and intragenic regions, especially for CGIs covering exons (34%, 505/1,471 in total, 502/1,449 on autosomes) (Figure 2A). Because the UCSC annotations underestimate the number of CGIs [16], we repeated this analysis with a less stringent custom CGI annotation and found a similar repartition of CGI methylation (Figure S2A,B in Additional file 1). We explored the relationship between CGI methylation in pre- and post-implantation stages and found that half of the CGIs methylated in post-implantation embryos already have persistent gametic methylation in blastocysts (Figure 2B). This reflects that CGIs that escape complete demethylation before implantation are more likely to reacquire methylation after implantation (Figure 2C). Consequently transient imprinted CGI methylation in blastocysts rarely translates into lifelong imprinted methylation [7,17]. We then monitored the kinetics of *de novo* methylation at CGIs and found that they acquire methylation at a slower rate compared with the bulk genome (Figure 2D). A similar delay in methylation is observed when we use our extended set of custom annotated CGIs (Figure S2C in Additional file 1). This delay is most evident at TSS-proximal CGIs (Figure 2E), as illustrated by the *Sycp3* promoter (Figure 2F) and several other promoters (Figure S3A in Additional file 1). Methylation of intergenic and intragenic CGIs is, on average, less delayed than at promoters (Figure 2E); nevertheless, many of these CGIs also gain delayed methylation as exemplified by intragenic CGIs in the *Bcl11b*, *Dact1* and *Cux1* genes (Figure 2F; Figure S3B in Additional file 1).

### CpG island methylation is recruited to important developmental genes

To gain insights into the function of CGI methylation, we performed ontology analyses on genes that gain CGI methylation after implantation. In accordance with previous data [7,11], promoter CGI methylation is enriched at genes involved in gamete functions (Figure 2G; Figure S4A in Additional file 1). Using our custom CGI annotation, we identified 87 CGI promoters with more than 50% methylation in E8.5 embryos and found that 79% (69/87) are associated with germline genes (Additional file 3). The targets identified here and in previous studies [7] reveal that promoter CGI methylation is recruited in particular to genes involved in gamete chromatin (*Brdt*, *H1fnt*, *Hist1h2aa/ba*, *H2afy3*), meiosis (*Spo11*, *Sycp1/2/3*, *Syce1/3*, *Msh4*, *Hormad1/2*) and the Piwi-interacting RNA (piRNA) pathway (*Piwil1/2/4*, *Mov10l1*, *Fkbp6*, *Mael*, *Tdrd1/9/12*, *Rnf17*, *Ddx4*, *Asz1*). In contrast, exon CGI methylation is targeted to genes with important developmental functions in the regulation of transcription, morphogenesis, signaling pathways and neuronal development (Figure 2G). As examples, key transcription factor genes (*Cux1*, *Bcl11b*, *Klf3*, *Daxx*, *Foxo3*, *Zfp64*) gain exon CGI methylation in the epiblast, as well as several genes of the Wnt/β-catenin signaling pathway that plays pivotal roles in embryogenesis and gastrulation. In contrast, we found no ontology category associated with intron CGI methylation (data not shown). To investigate if intragenic CGI methylation correlates with transcription of the surrounding gene as is the case in oocytes [4,18], we compared CGI methylation with RNA-Seq in E8.5 embryos and found a tendency for methylated CGIs in exons, but not introns, to be located within active transcription units (Figure S4B in Additional file 1). To ask if CGI methylation persists in the adult, we interrogated a published dataset from mouse adult tissues [3] and found that CGIs in promoters, exons, introns or intergenic regions remain highly methylated in all tissues (Figure 2H). Thus, CGI methylation in the epiblast is targeted to important developmental genes and constitutes a stable epigenetic signature of all somatic lineages.

### A class of CpG islands gains partial methylation in somatic lineages

**Figure 3:**
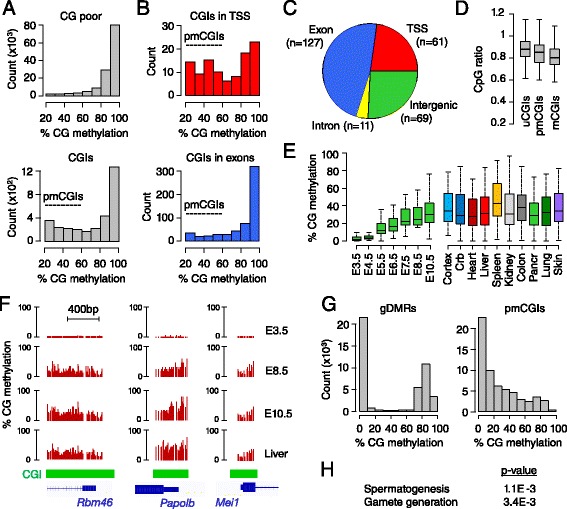
**A class of CpG islands gains partial methylation during development. (A)** Density histograms showing the distribution of methylation in E8.5 embryos in genomic tiles (400 bp) covering CpG-poor regions and CGIs, which reveals a high prevalence of partially methylated CGIs (pmCGIs). **(B)** Frequency of pmCGIs in CGIs covering TSSs and exons. **(C)** Distribution of pmCGIs in TSSs, exons, introns and intergenic regions. **(D)** Comparison of the distribution of CpG ratios in unmethylated (u; <10% methylation in E8.5 embryos), partially methylated (pm; >15% and <60% methylation) and methylated (m; >60% methylation) CGIs. **(E)** Dynamics of DNA methylation at TSS-proximal pmCGIs (defined as >15% and <60% methylation in E8.5 embryos) across embryonic development and in adult tissues. Crb, cerebellum; Pancr, pancreas. **(F)** Examples of RRBS methylation scores at three TSS-associated pmCGIs in blastocysts (E3.5), post-implantation embryos (E8.5 and E10.5) and adult liver. **(G)** Distribution of RRBS single-allele methylation scores from E8.5 embryos in TSS-proximal pmCGIs compared with imprinted gDMRs. **(H)** Gene Ontology terms associated with TSS-proximal pmCGIs.

We noted that CGIs have a peculiar distribution of CpG methylation in post-implantation embryos characterized by a high prevalence of intermediate methylation (Figure 3A). We defined partially methylated CGIs (pmCGIs) as having methylation of between 15 and 60% in E8.5 embryos (excluding the known imprinted differentially methylated regions (DMRs) and the X chromosome). The pmCGIs are found within and outside genes but their relative frequency is the highest in TSSs (Figure 3B,C). Interestingly their CpG ratio is intermediate between those of methylated and unmethylated CGIs (Figure 3D). To investigate the possibility that the pmCGIs gain methylation at later stages of development, we generated RRBS methylomes in E10.5 embryos and adult liver, and investigated methylomes from adult tissues [3]. Most promoter pmCGIs retain partial methylation throughout development and in adult tissues (Figure 3E,F). Similarly, a high proportion of intergenic and intragenic pmCGI harbors partial methylation in all tissues while some occasionally are fully methylated in some tissues (data not shown). We then asked if this partial methylation represents an allele-specific methylation or a low methylation per allele. To this end we extracted single-allele methylation data from the sequencing reads and found that, in contrast to the allele-specific methylation of imprinted gDMRs, the partial methylation at pmCGIs results from a low density of methylated cytosines per allele (Figure 3G). This is confirmed by bisulfite cloning and sequencing of larger amplicons (400 to 500 bp) in four pmCGI promoters in adult liver (Additional file 1). Interestingly, whole-genome bisulfite sequencing and the bisulfite cloning reveal that pmCGIs contain patches of low and high susceptibility to methylation (Additional file 1), which could reflect differential susceptibility caused by the positioning of nucleosomes. To further characterize the promoter pmCGIs, we performed an ontology analysis and found that, similar to the fully methylated CGI promoters, they are enriched for germline-specific genes (Figure 3H). Using our custom annotation pipeline, we identified 50 high confidence pmCGI promoters and counted that 60% (30/50) are linked to germline genes (Additional file 3). Representative examples include *Smc1b*, *Papolb*, *Boll*, *Mei1*, *Rbmxl2* and *Rbm46* (Figure 3F). This identifies a novel class of methylated promoter CGIs and extends the repertoire of germline genes targeted by DNA methylation in embryogenesis.

### DNMT3A and DNMT3B cooperate to methylate the genome

**Figure 4:**
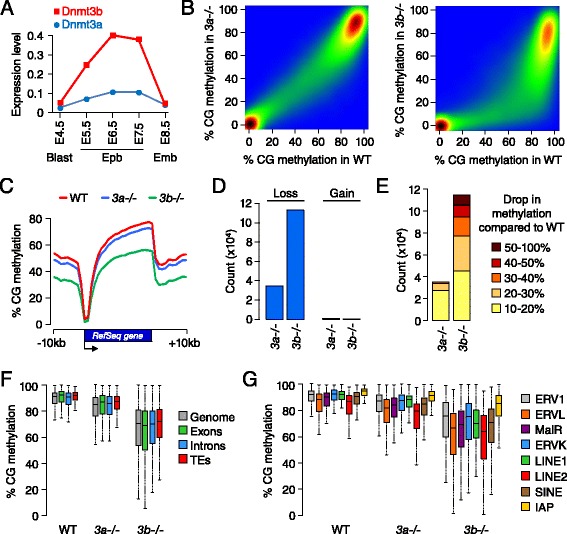
**Methylome profiling in DNMT3A and DNMT3B**-**deficient embryos. (A)** mRNA expression of *Dnmt3a/b* genes in embryos. Expression was measured by RT-qPCR on 5 to 10 pooled embryos and is depicted as a ratio relative to the expression of two housekeeping genes (*Actb* and *Rpl13a*). The primers used for RT-qPCR were designed in the last exons to amplify all isoforms. Blast, blastocyst; Epb, epiblast; Emb, embryo. **(B)** Pairwise comparison of CpG methylation in 400 bp tiles in wild-type (WT) compared with *Dnmt3a*-*/*- and *Dnmt3b*-*/*- E8.5 embryos. **(C)** Distribution of CpG methylation in RefSeq genes and 10 kb of flanking sequences in WT, *Dnmt3a*-*/*- or *Dnmt3b*-*/*- E8.5 embryos. **(D)** Number of 400 bp tiles that lose or gain more than 10% methylation in *Dnmt3a*-*/*- and *Dnmt3b*-*/*- compared with WT E8.5 embryos. **(E)** Detailed representation of the extent of methylation loss detected in 400 bp tiles in *Dnmt3a*-*/*- and *Dnmt3b*-*/*- compared with WT E8.5 embryos. **(F)** Boxplot representing the distribution of CpG methylation in the whole genome (400 bp tiles), exons, introns and transposable elements (TEs) in WT, *Dnmt3a*-*/*- and *Dnmt3b*-*/*- E8.5 embryos. Only sequences with >50% methylation in WT E8.5 embryos are considered. **(G)** Distribution of CpG methylation in several families of transposable elements in WT, *Dnmt3a*-*/*- and *Dnmt3b*-*/*- E8.5 embryos. IAP, intracisternal A-particle; LINE, long interspersed nuclear element; SINE, short interspersed nuclear element.

Next we investigated the contribution of the *de novo* enzymes DNMT3A and DNMT3B to methylation in embryos. We first followed the expression of *Dnmt3a/b* mRNAs by quantitative RT-PCR (RT-qPCR) and found that both genes are upregulated in early epiblast cells, which coincides with the onset of genome-wide methylation (Figure 4A). Notably, *Dnmt3b* mRNAs reach higher levels of expression than *Dnmt3a* (Figure 4A). RNA-Seq indicates that embryos express predominantly the short *Dnmt3a2* isoform and the full length *Dnmt3b1* isoform (Figure S6A in Additional file 1). We generated embryos homozygous for catalytically inactive alleles of *Dnmt3a* and *Dnmt3b* (referred to as *Dnmt3a*-*/*- and *Dnmt3b*-*/*-) and performed RRBS at the E8.5 stage, which led to highly reproducible data in independent embryos (Figure S1E in Additional file 1). Importantly, we verified that the inactivation of one *Dnmt3* gene does not modify the expression of the other *Dnmt* genes in embryos (Figure S6B,C in Additional file 1). We found that the inactivation of *Dnmt3a* or *Dnmt3b* leads to a partial reduction in global methylation, indicating that the inactivation of one enzyme is compensated for by the other and that both enzymes cooperate to methylate the bulk genome (Figure 4B,C). The decrease in methylation is unidirectional with no signs of gain of methylation, confirming that these enzymes solely act as methylases (Figure 4D). Overall, the inactivation of *Dnmt3b* leads to a higher number of hypomethylated sequences and an increased amplitude in the loss of methylation compared with *Dnmt3a* (Figure 4E). Detailed quantification is given in Figure 4F and shows that the median methylation of methylated sequences in E8.5 embryos drops from 91% in wild type (WT) to 86% in *Dnmt3a*-*/*- and 72% in *Dnmt3b*-*/*- embryos. These variations are equally distributed in exons, introns and transposable elements (Figure 4F). We also monitored methylation of various classes of transposable elements (long interspersed nuclear elements (LINEs), short interspersed nuclear element (SINEs), long terminal repeats) and found that they follow the same trend with a small decrease in methylation (approximately 5%) in *Dnmt3a*-*/*- embryos and a more pronounced decrease (approximately 20%) in *Dnmt3b*-*/*- embryos (Figure 4G). The exception is intracisternal A-particle elements, which are marginally affected in *Dnmt3a*-*/*- and *Dnmt3b*-*/*- embryos, which is consistent with previous data [10,14] and reflects that intracisternal A-particles maintain high methylation in pre-implantation stages. We conclude that DNMT3A and DNMT3B cooperate to establish DNA methylation in embryos, with DNMT3B having a greater contribution than DNMT3A.

### CpG islands are preferentially methylated by DNMT3B

**Figure 5:**
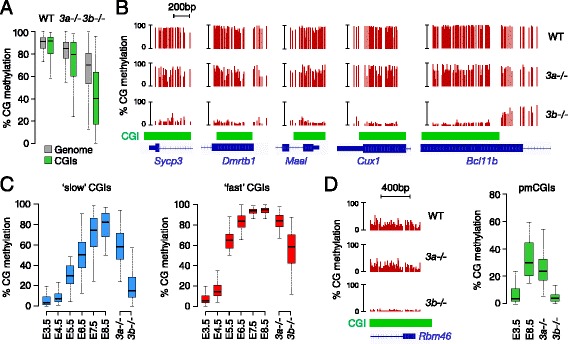
**DNMT3B methylates CpG islands in epiblast cells. (A)** Boxplot showing the distribution of CpG methylation in the whole genome (400 bp tiles) and CGIs in WT, *Dnmt3a*-*/*- and *Dnmt3b*-*/*- E8.5 embryos. Only sequences with >50% methylation in WT E8.5 embryos are considered. **(B)** Representative examples of RRBS profiles at DNMT3B-dependent CGIs in WT, *Dnmt3a*-*/*- and *Dnmt3b*-*/*- E8.5 embryos in gene promoters (*Sycp3*, *Dmrtb1*, *Mael*) and gene bodies (*Cux1*, chr5:136,274,862-136,275,397; *Bcl11b*, chr12:107,915,284-107,917,294). **(C)** Dynamics of DNA methylation across embryonic development and in *Dnmt3*-mutant E8.5 embryos in slow (<20% methylation in E3.5, <50% in E5.5 and >50% in E8.5) and fast (<20% methylation in E3.5, >50% methylation in E5.5 and >50% methylation in E8.5) CGIs. **(D)** DNMT3B methylates pmCGIs, as illustrated by the RRBS profiles in E8.5 embryos at the *Rbm46* CGI. The box plot shows the distribution of methylation at all pmCGIs in blastocysts (E3.5), WT E8.5 embryos and *Dnmt3*-mutant E8.5 embryos.

We next investigated if DNMT3A and DNMT3B have specific targets. As shown in Figure 4E, we identified severely hypomethylated sequences in *Dnmt3b*-/- but not *Dnmt3a*-/- embryos, indicating that only DNMT3B has specific targets for methylation. We identified 1,759 *Dnmt3b*-dependent targets defined as losing more than 60% methylation in *Dnmt3b*-*/*- compared with WT embryos (Additional file 4). These targets are distributed in promoters, gene bodies and intergenic regions (Figure S7A in Additional file 1) and have an increased CpG density (Figure S7B in Additional file 1), suggesting a preferential role for DNMT3B at CGIs. To verify this hypothesis, we monitored methylation at CGIs and found that they are more severely demethylated in *Dnmt3b*-/- embryos compared with the bulk genome (Figure 5A). Many CGIs are markedly hypomethylated in *Dnmt3b*-/- compared with WT embryos, such as in the promoters of *Sycp3*, *Dmrtb1*, *Mael*, and gene bodies of *Cux1* and *Bcl11b* (Figure 5B). Remarkably, the DNMT3B-dependent CGIs overlap with the ones that acquire delayed methylation in late epiblast cells (for example, *Sycp3* and *Bcl11b* in Figures 2F and 5B). We investigated this observation on a global scale and confirmed that the methylation of the ‘slow’ CGIs returns to levels close to those of blastocysts in *Dnmt3b*-*/*- E8.5 embryos (Figure 5C, left panel). In contrast the methylation of the ‘fast’ CGIs is less affected by the inactivation of DNMT3B and can be partly compensated for by DNMT3A (Figure 5C, right panel). The methylation of the pmCGIs, which can be viewed as extreme cases of ‘slow’ CGIs, also strictly depends on DNMT3B activity (Figure 5D). Lastly, we show that the DNMT3B-dependent targets identified in E8.5 embryos are hypomethylated to a similar extent in limbs from E11.5 *Dnmt3b*-*/*- embryos (Figure S7C in Additional file 1), demonstrating that DNMT3A is incapable of compensating DNMT3B at these targets even over a prolonged period of development. Altogether this reveals a specific role for DNMT3B in the methylation of CpG-rich DNA in epiblast cells.

### Influence of DNMT3B-dependent methylation on gene expression in embryos

**Figure 6:**
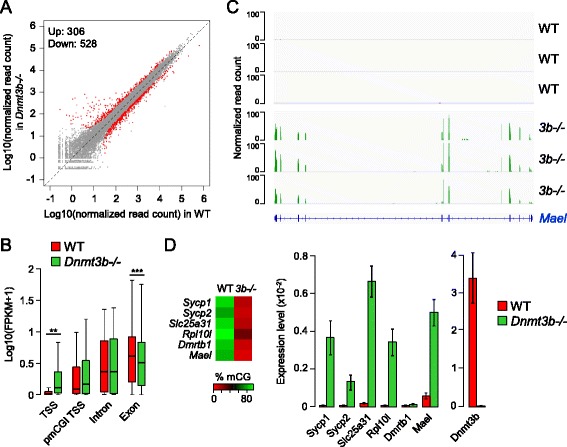
**Transcriptome of** ***Dnmt3b***-*/*- **embryos. (A)** Scatter plot of normalized read counts per gene (average of three replicates) calculated by DESeq2 in WT and *Dnmt3b*-*/*- E8.5 embryos. The red dots represent the genes called differentially expressed with a false discovery rate-adjusted *P*-value <0.05 and fold change >2. The numbers of genes upregulated and downregulated are indicated. **(B)** Consequences of reduced CGI methylation on gene expression. The boxplot shows Fragments Per Kilobase of exon per Million fragments mapped (FPKM) scores for genes with reduced methylation (>50% decrease of methylation in *Dnmt3b*-*/*- compared with WT embryos) of a CGI covering a TSS, exon or intron, as well as genes with a pmCGI in the TSS. **P < 0.01, ***P < 0.001 (Wilcoxon test). **(C)** RNA-Seq read coverage at the germline-specific gene *Mael* in three WT and *Dnmt3b*-*/*- embryos. **(D)** RT-qPCR validation of the upregulation of germline genes in *Dnmt3b*-*/*- E8.5 embryos. The heatmap on the left depicts the extent of TSS CGI hypomethylation in *Dnmt3b*-*/*- embryos. Gene expression is depicted in the bar graphs as a ratio relative to the expression of two housekeeping genes (*Actb* and *Rpl13a*). The error bars represent mean deviations from measurements in independent embryos (n = 3). As a control we show the expression of *Dnmt3b* measured with primers that amplify within the Cre-deleted catalytic exons.

To explore the influence of DNMT3B-dependent methylation on gene transcription, we conducted RNA-Seq in three WT and *Dnmt3b*-*/*- E8.5 embryos (Figure S6D in Additional file 1). Overall we found relatively similar transcriptome profiles (Figure 6A). We identified 306 upregulated and 528 downregulated genes in *Dnmt3b*-*/*- compared with WT embryos, but genes with the highest fold change are mostly upregulated (Figure 6A; Additional file 5). The reduction of promoter CGI methylation in *Dnmt3b*-*/*- embryos strongly correlates with gene upregulation (Figure 6B), which leads to the ectopic activation of many full length germline transcripts that constitute 81% of the genes upregulated more than five-fold in *Dnmt3b*-*/*- embryos (Figure 6C; Figure S8A,B in Additional file 1). The only other genes strongly upregulated in *Dnm3b*-*/*- embryos are genes of the *Rhox* cluster (*Gm9*, *Rhox4g*, *Rhox4e*, *Rhox9*) [14] and members of a family of X-linked imprinted genes (*Xlr3a/b/c*, *Xlr4a/b/c*) (Figure S8A in Additional file 1; Additional file 5). Thus, DNMT3B represses a small number of genes mainly associated with germline function. We validated the potent upregulation of germline genes by RT-qPCR in E8.5 *Dnmt3b*-*/*- embryos as well as limbs from E11.5 *Dnmt3b*-*/*- embryos (Figure 6D; Figure S8C in Additional file 1), demonstrating that CGI methylation establishes long-term silencing of germline promoters throughout development. Interestingly, the absence of partial methylation at pmCGI promoters is also associated with a minor increase of transcript abundance in *Dnmt3b*-*/*- embryos (Figure 6B), which was verified by RT-qPCR at three germline pmCGI genes (Figure S8C in Additional file 1). Thus, partial promoter methylation has a small contribution to promoter silencing at some genes. Consistent with a contribution of DNA methylation to the silencing of pluripotency genes in development [19], reduced promoter methylation is associated with a modest increase in transcript abundance of several pluripotency genes in *Dnmt3b*-*/*- embryos, in particular *Dppa3/Stella* and *Dppa4* (Figure S8D in Additional file 1). Finally, we investigated the influence of intragenic CGI methylation on transcript abundance. Interestingly, RNA-Seq indicates that genes with reduced CGI methylation over an exon are significantly downregulated in *Dnmt3b*-*/*- embryos (Figure 6B), consistent with a role of intragenic CGI methylation in influencing the expression of the surrounding gene [20]. Overall, this shows that DNMT3B-dependent CGI methylation has a repressive function at promoters and a putative positive influence on transcript abundance in the body of genes.

### DNMT3 knockouts distinguish germline from somatic imprinted differentially methylated regions

**Figure 7:**
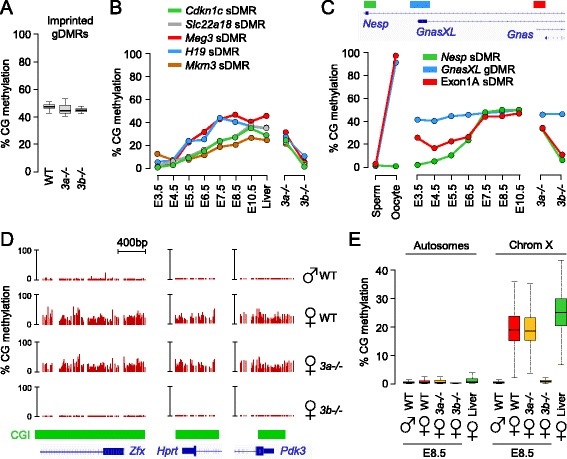
**Role of DNMT3B at imprinted differentially methylated regions and X-linked CpG islands in embryos. (A)** Box-plot representing the methylation of 17 imprinted germline DMRs in WT, *Dnmt3a*-*/*- and *Dnmt3b*-*/*- E8.5 embryos. **(B)** Dynamics of methylation throughout development and in *Dnmt3* knockout E8.5 embryos at somatic DMRs: *Cdkn1c* (chr7:143,459,734-143,460,383), *Slc22a18* (chr7:143,465,018-143,465,543), *Meg3* (chr12:109,540,809-109,541,073), *H19* (chr7:142,578,145-142,578,462) and *Mkrn3* (chr7:62,419,498-62,420,497). **(C)** Dynamics of methylation throughout development and in *Dnmt3* knockout E8.5 embryos at the three DMRs of the *Gnas* locus. The genomic organization of the locus and the position of the DMRs are shown on top of the graph. The detailed RRBS profiles are shown in Figure S9B in Additional file 1. **(D)** Examples of RRBS profiles at CGI promoters of three X-inactivated genes in female WT and *Dnmt3*-mutant embryos at the E8.5 stage. **(E)** Box plots of the global distribution of CGI methylation in autosomes (left) and on the X chromosome (right) in WT E8.5 embryos, female *Dnmt3*-mutant embryos and female adult liver.

It has been suggested that the DNMT3 enzymes participate in the maintenance of DNA methylation in mammalian cells [21]. To ask if DNMT3A/B contribute to the maintenance of DNA methylation imprints, we monitored methylation at 17 imprinted gDMRs and found no signs of hypomethylation in *Dnmt3a*-*/*- or *Dnmt3b*-*/*- embryos (Figure 7A; Figure S9A in Additional file 1). This agrees with previous results obtained at the *Igf2r* gDMR [14] and two paternal gDMRs [22], and indicates that the individual DNMT3 enzymes are dispensable for the maintenance of methylation imprints *in vivo*. The possibility remains that DNMT3A/B are redundant for the maintenance of DNA methylation, which needs to be tested in double mutants. In contrast to gDMRs, secondary somatic DMRs (sDMRs) acquire allele-specific DNA methylation after implantation. We profiled DNA methylation at several known sDMRs and observed that, in accordance with published data [23,24], they acquire methylation with variable kinetics in development (Figure 7B). Interestingly, these sDMRs all acquire methylation in a DNMT3B-dependent manner (Figure 7B). Accordingly, this is associated with a less than two-fold increase of some of these imprinted transcripts (*H19*, *Meg3*, *Cdkn1c*, *Mkrn3*) in *Dnmt3b*-*/*- embryos (Figure S8E in Additional file 1). We then reasoned that our data can serve to accurately distinguish gDMRs from sDMRs: gDMRs are stable throughout embryogenesis and unaffected in *Dnmt3* mutants, whereas sDMRs gain DNMT3B-dependent methylation after implantation. Using these criteria, we reassessed the known gDMRs and confirmed that they inherit stable gametic methylation except for the *Exon1A* gDMR at the *Gnas* locus. The *Gnas* locus is unusual as previous studies identified two gDMRs in the *GnasXL* promoter and *Exon1A* (Figure 7C) [25,26]. We now reveal that the *Exon1A* DMR is a sDMR: it inefficiently maintains maternal methylation in blastocysts and undergoes DNMT3B-dependent *de novo* methylation after implantation (Figure 7C; Figure S9B in Additional file 1). This suggests a revised model of imprinting at the *Gnas* locus under the control of only one gDMR.

### DNMT3B methylates CpG islands on the inactive X chromosome in females

CGIs gain DNA methylation on the inactive copy of the X chromosome in female XX embryos to stabilize X-inactivation. To explore the role of DNMT3 enzymes in X-linked CGI methylation, we examined RRBS methylomes in female embryos. A visual inspection at promoters of X-inactivated genes shows that they acquire partial CGI methylation in female but not male E8.5 embryos (Figure 7D). We investigated this in a systematic way and found that all the CGIs on the X chromosome show a concordant gain of methylation in female embryos (Figure 7E). The methylation in female E8.5 embryos is only slightly lower than the one observed in a female adult liver (Figure 7E), indicating that most of the X-linked CGI methylation is already established in early post-implantation embryos. In accordance with human data [27], we found that X-linked CGI methylation is not restricted to promoters but occurs at all CGIs in promoters, gene bodies and intergenic regions (Figure S10A in Additional file 1). In contrast, CGI methylation is not found at some of the genes known to escape X-inactivation in the mouse (Figure S10B in Additional file 1). Out of 13 described escapee genes in the mouse [28], 10 have their promoter covered in our dataset and 5 show no signs of promoter methylation (Figure S10C in Additional file 1). The other five genes might be mis-annotated escapees or could have alternative promoters. We then investigated the contribution of DNMT3 enzymes to X-linked methylation by examining RRBS methylomes generated in *Dnmt3a*-*/*- and *Dnmt3b*-*/*- female E8.5 embryos. Strikingly, X-linked CGI methylation is unaffected in *Dnmt3a*-*/*- embryos but completely absent in *Dnmt3b*-*/*- embryos (Figure 7D,E). DNMT3B methylates all the CGIs of the inactive X chromosome in promoters but also intragenic and intergenic regions (Figure S10A in Additional file 1). These data extend results obtained on candidate X-linked CGIs [29,30] and demonstrate that DNMT3B catalyzes methylation of all CGIs on the inactive X chromosome in female embryos.

## Discussion

Using quantitative profiling of cytosine methylation at single-base resolution, we determined the kinetics of DNA methylation and the specificity of the *de novo* DNMT3 enzymes in mouse embryos *in vivo*. This extends previous findings on the dynamics of DNA methylation in mouse embryogenesis [4-8]. Our data provide a useful resource for investigating the inheritance and reprogramming of DNA methylation in embryos, notably by accurately distinguishing gDMRs from sDMRs at imprinted loci. We reassessed all the known gDMRs at imprinted loci and confirmed that they inherit gametic DNA methylation with no contribution of *de novo* methylation after fertilization. The exception is the *Exon1A* DMR at the *Gnas* locus, which was previously identified as a gDMR that maintains maternal methylation in blastocysts and post-implantation embryos [25]. Another gDMR was described in the *GnasXL* promoter, which made the *Gnas* locus an unusual case of imprinted loci with two gDMRs. We now reveal that the *Exon1A* DMR is only partially resistant to methylation reprogramming in blastocysts and gains DNMT3B-dependent methylation after implantation, which classifies it as a sDMR. This suggests a revised model of imprinting at the *Gnas* locus under the control of only one gDMR covering the *GnasXL* promoter, which then controls the establishment of the sDMRs *in cis*. In accordance with this model, the deletion of the *GnasXL* DMR influences methylation of the *Exon1A* DMR in embryos, whereas the opposite is not true [26,31,32].

We show that the embryo acquires global DNA methylation within a short period of time between 4.5 and 6.5 days *post coitum* in epiblast cells. This is faster than when it is recapitulated *in vitro* in embryonic stem (ES) cells switched from 2i to serum conditions. In this case, bisulfite sequencing profiling revealed that global methylation is completed only 5 to 7 days after adapting ES cells from 2i to serum [33]. These differences could be due to different levels of expression of the DNMTs. One important question is whether *de novo* methylation occurs by default or is triggered by pre-existing factors to specific sites in the genome. Our kinetic study supports the default model of methylation because (1) methylation increases with similar kinetics throughout the genome, and (2) methylation increases rapidly as soon as DNMT3 genes are upregulated in early epiblast cells. As we studied whole populations of cells, however, we cannot exclude the existence of cell-to-cell heterogeneity in the kinetics of acquisition. This hints at a random mechanism of deposition of methylation rather than initiation and spreading from defined methylation centers, as has also been observed in ES cells switched from 2i to serum [33]. This model is consistent with studies showing that DNMT3 enzymes are recruited by default to chromatin via their PWWP domain [34-36].

Our results highlight the distinct regulation of DNA methylation at CGIs compared with the bulk genome: CGIs are mostly resistant to methylation at implantation, acquire delayed methylation in late epiblast cells, and frequently acquire partial methylation. Only 5% (713/14,085) of the annotated UCSC CGIs gain more than 50% methylation in the post-implantation embryo. The rarity of methylation at CGIs suggests that they are intrinsically protected from default methylation at the time of implantation. This could be mediated by proteins with CXXC domains that recognize stretches of CpG-rich DNA and could impose a chromatin structure that makes DNA refractory to methylation. The methylation-free state of CGIs could also be related to their activity as platforms for the binding of transcription factors. In line with this idea, mutations in transcription factor binding sites can relieve the protection from methylation at CGIs [37,38]. This model would predict that all CGIs are transcriptionally active or at least bound by the transcription machinery in embryos at the time of *de novo* methylation. An alternative possibility is that CGIs are ‘methylatable’ but that methylation is constantly removed via demethylation. The maintenance of the hypomethylated state of CGIs by demethylation could involve the action of TDG (thymine DNA glycosylase) or TET (ten-eleven translocation) proteins. Indeed, the inactivation of TDG in the mouse or the combined deficiency of TET1/2/3 in mouse ES cells leads to the hypermethylation of some CGIs and impairs developmental potential [39-41]. In this context, the methylation of a subset of CGIs probably requires specific molecular pathways necessary to override the intrinsic resistance to methylation before allowing the deposition of DNA methylation. This would explain that CGI methylation is delayed compared with the bulk genome and can be partial due to inefficient targeting. To date very little is known about these molecular mechanisms that recruit DNA methylation to a subset of CGIs. Existing evidence indicates that it could involve non-coding RNAs [42] or DNA-binding repressors such as E2F6 [43].

Interestingly, intragenic and intergenic CGIs are much more prone to developmental DNA methylation than in the TSS. This was observed previously in differentiated lineages and suggests important roles for intragenic methylation in gene regulation [16,44,45]. In line with this idea, we show that intragenic CGI methylation is recruited to genes with important developmental functions in embryonic morphogenesis, signaling pathways and transcription regulation. In addition, our RNA-Seq data show that reduced intragenic CGI methylation in *Dnmt3b*-*/*- embryos is associated with small changes in transcript abundance of the surrounding genes, suggesting that intragenic CGI methylation plays a role in the fine tuning of expression of developmental genes in the embryo. A positive correlation between intragenic DNA methylation and gene expression has been documented previously in normal and malignant mammalian cells (reviewed in [20]). Functionally, it can be speculated that intragenic methylation facilitates the transcription elongation or influences the posttranscriptional processing of the surrounding mRNA. Another possibility is that DNA methylation regulates the activity of alternative intragenic CGI promoters or promoters of regulatory non-coding RNAs. However, we found so far little evidence in the RNA-Seq data for activated transcription in the sense or antisense orientation at demethylated intragenic CGIs in *Dnmt3b*-*/*- embryos (data not shown). Hence additional investigations are needed to explore the possible functional impact of intragenic CGI methylation in gene regulation [20]. In the TSS, CGI methylation is extremely rare (less than 1% have more than 20% methylation) and occurs almost exclusively at the promoters of germline-specific genes. This remarkable specificity, together with the fact that germline genes are durably reactivated in methylation-deficient embryos and cultured cells as shown by us and others [11,46-48], indicates that the silencing of the germline program in soma is a major evolutionary function of DNA methylation in mammals.

Mammalian genomes encode two *de novo* methyltransferases (DNMT3A and DNMT3B) but their respective contribution to embryonic methylation remained poorly characterized. We generated methylomes in embryos with catalytically inactive mutants of *Dnmt3a* or *Dnmt3b* and found that, in agreement with data at candidate loci [7,10,14,29,43], DNMT3A and DNMT3B have both redundant and specific functions. First, we show that the inactivation of *Dnmt3a* or *Dnmt3b* induces a partial hypomethylation of the genome. The sum of the methylation in the knockout embryos is much higher than the methylation in WT embryos, indicating that the catalytic activities of both enzymes can compensate for each other and cooperate to methylate the genome. We note, however, that DNMT3B makes a greater contribution than DNMT3A, probably because of the higher expression of *Dnmt3b* compared with *Dnmt3a* in epiblasts as observed here by RT-qPCR (Figure 4A) and previously by immunostaining [10,49]. Their redundant function might have evolved to ensure robust and efficient methylation in embryos. Despite the global redundancy, DNMT3B also has a specific role in the methylation of many CGIs on autosomes and the inactive X chromosome that are dramatically hypomethylated in *Dnmt3b*-*/*- embryos. This highlights again that DNA methylation at CGIs is controlled by different molecular pathways compared with the bulk genome, which involve the preferential recruitment of DNMT3B. Our data contribute to explain the more severe phenotype of *Dnmt3b*-*/*- compared with *Dnmt3a*-*/*- mice [10]. DNMT3B makes a greater contribution to genome methylation and specifically methylates a set of CpG-dense sequences associated with developmental and imprinted genes, which leads to the deregulation of germline, pluripotency and imprinted genes and hundreds of other direct or indirect targets in *Dnmt3b*-deficient embryos. Which of these genes contribute the most to the embryonic lethality of *Dnmt3b*-*/*- animals remains to be investigated. The preferential role of DNMT3B at CGIs is compatible with data showing that mice or human ICF syndrome (immunodeficiency, centromeric instability, facial anomalies) patients with DNMT3B mutations have reduced methylation at CGIs on autosomes and the inactive X chromosome [43,50,51], suggesting a functional conservation of DNMT3B function between mice and humans. Our findings also shed new light on the possible molecular dysfunctions caused by the mutations of DNMT3B in the human ICF syndrome and might help to identify new epigenetically deregulated targets for diagnosis [52].

## Conclusions

We revealed the target specificities of the *de novo* methyltransferases DNMT3A and DNMT3B in mouse development *in vivo*. We show that they have redundant catalytic functions in global genome methylation at implantation, and that DNMT3B specifically methylates a defined set of CpG islands on autosomes and on the X chromosome. This indicates that DNMT3 enzymes evolved to play both redundant and specific functions in mammalian embryos. Further studies are required to elucidate the molecular mechanisms responsible for the recruitment of DNMT3B activity to CpG-dense regions, which might identify new pathways inducing abnormal CGI methylation in cancer.

## Materials and methods

### Biological samples

All embryos were obtained from naturally mated C57BL/6 mice. We designated the morning of the vaginal plug as E0.5 and performed all the dissections at the same hour of the day (1 pm). Blastocysts (E3.5 to E4.5) were collected by flushing the uteri with M2 medium. After implantation, we manually dissected individual embryos in M2 medium. At E5.5 to E7.5 we separated the epiblast from the extraembryonic tissues by manual dissection. We dissected whole embryos at E8.5 to E10.5, and dissected forelimb buds from E11.5 embryos. Sperm was isolated from the caput epididymis of adult CD-1 mice. We prepared genomic DNA by proteinase K digestion, phenol/chloroform extraction and precipitation with ethanol. To generate catalytically inactive mutants of *Dnmt3* genes, we crossed *Dnmt3a*-2lox [53] and *Dnmt3b*-2lox [54] mice (provided by the Massachusetts General Hospital, Boston, MA, USA) with a C57BL/6 ACTB-Cre deleter line [55] (provided by the Institut Clinique de la Souris, Illkirch, France). *Dnmt3* knockout embryos were then obtained by mating heterozygous males and females.

### Preparation of RRBS libraries

We prepared RRBS libraries from 100 pooled E3.5 blastocysts, 50 pooled E4.5 blastocysts, 25 pooled E5.5 epiblasts, 15 pooled E6.5 epiblasts, and 10 pooled E7.5 epiblasts. At E8.5, we prepared RRBS libraries from a pool of embryos as well as two individual WT, *Dnmt3a*-*/*- and *Dnmt3b*-*/*- embryos. At E10.5, we prepared RRBS libraries from pools of WT embryos. At E11.5 we prepared RRBS libraries from limbs of two WT and two *Dnmt3b*-*/*- embryos. RRBS libraries were prepared according to a published protocol [56] with modifications. Briefly, we digested 25 to 100 ng of genomic DNA for 5 h with *Msp*I (Thermo Scientific, Waltham, MA, USA) followed by end-repair, A-tailing (with Klenow fragment, Thermo Scientific) and ligation to paired-end methylated adapters (with T4 DNA ligase, Thermo Scientific) in Tango 1X buffer. We purified fragments in the range 150 to 400 bp (insert plus adapter size) by electrophoresis on a 3% (w/v) agarose 0.5X TBE gel with the MinElute gel extraction kit (Qiagen). We then performed two rounds of bisulfite conversion with the EpiTect kit (Qiagen) according to the manufacturer’s instructions. Final RRBS libraries were PCR amplified with PfUTurbo Cx hotstart DNA polymerase (Agilent, Santa Clara, CA, USA) and indexed PE Illumina primers using the following PCR conditions: 95°C for 2 minutes, 14 to 16 cycles (95°C for 30 s, 65°C for 30 s, 72°C for 45 s), 72°C for 7 minutes. The libraries were purified with AMPure magnetic beads (Beckman Coulter, Brea, CA, USA), quantified with a Qubit fluorometer (Life Technologies, Carlsbad, CA, USA) and verified by loading 10 ng of the library on a 4-20% Criterion precast polyacrylamide gel (Biorad, Hercules, CA, USA) stained with SYBR Green. The libraries were paired-end sequenced (2 × 75 bp) in multiplex on an Illumina HiSeq2000 by Integragen SA (Evry, France) to generate an average of 30 million pairs of reads per sample (Figure S1A in Additional file 1).

### Processing of RRBS sequencing reads

We performed quality control checks on sequencing reads with FastQC [57]. Reads were trimmed with Trim Galore (v0.2.1) [58] to remove adapter sequences and low-quality ends with a Phred score below 20. Trim Galore was run in --rrbs mode to remove two additional bases artificially introduced at the *Msp*I restriction sites. We aligned sequencing reads to the mouse mm10 genome with BSMAP (v2.74) [59] using the RRBS mode. For the mapping, we allowed a maximum of two mismatches and an insertion size for paired-end sequences of between 30 and 400 bp. We extracted methylation scores as the ratio of the number of Cs over the total number of Cs and Ts. We combined CpG methylation ratios from both strands and filtered for a minimum sequencing depth of 8×. We estimated the bisulfite conversion efficiency by calculating the C to T conversion at non-CpG sites, which was in most cases greater than 99.5% (Figure S1A in Additional file 1). Methylation scores were visualized with the IGV browser [60].

### RRBS data analysis

RefSeq genes, transposons and CGI annotations were retrieved from the UCSC mm10 annotation. We filtered transposons to have a minimum size of 200 bp. HCPs (high CpG promoters), ICPs (intermediate CpG promoters) and LCPs (low CpG promoters) were annotated as previously described [19]. The genomic coordinates of the imprinted DMRs were retrieved from the Wamidex imprinting database [61]. The genomic coordinates of canyons were retrieved from a published dataset [15]. For oocyte methylation, we retrieved a published RRBS dataset (GSE34864) [6] and averaged the methylation scores from all the oocyte replicates in the dataset. To annotate CGIs, we measured the distance between the middle of the CGI and the closest RefSeq TSS and overlapped CGIs with RefSeq exons and introns. We defined the CGI categories as follows: TSS, the CGI is less than 1,000 bp from a TSS; exon, the CGI is more than 1,000 bp from a TSS and overlaps at least 1 bp of an exon; intron, the CGI is more than 1,000 bp from a TSS and is entirely included in an intron; intergenic, the CGI does not meet any of the previous criteria. To follow the global dynamics at the genome level, we averaged methylation in 400 bp tiles containing at least three CpGs. To identify DNMT3B-dependent targets, we selected genomic tiles with a difference in methylation (WT minus *Dnmt3b*-*/*-) greater than 60%, and then merged overlapping tiles. To analyze methylation from single alleles, we mapped sequencing reads with Bismark [62], which returns mapping information on single reads. We then processed the Bismark output to extract methylation scores of individual sequenced molecules. We performed gene ontology analysis using the DAVID functional annotation tool [63]. All data processing and representation were performed with the R software using custom developed scripts.

### Custom CGI annotation

To generate our custom CGI annotation, we split the genome into 150 bp sliding windows with a 25 bp offset and selected windows with a GC percentage greater than 55% and a CpG ratio (observed/expected) greater than 0.65. We then merged the windows closer than 50 bp and selected the windows larger than 250 bp. To identify high-confidence custom promoter CGIs (Additional file 3), we used the same procedure with a minimal size of 225 bp, and then filtered the custom CGIs to be less than 800 bp from a RefSeq TSS.

### Bisulfite sequencing and cloning

Bisulfite conversion of genomic DNA was performed with the Epitect kit (Qiagen). We performed PCR amplification of converted DNA followed by cloning as described [7]. Cloned PCR products were sequenced, aligned with the BISMA software [64] and filtered to remove clonal biases. The sequences of the primers are provided in the Additional file 6.

### RNA-Seq

We prepared RNA-Seq libraries from three WT and *Dnmt3b*-*/*- littermate embryos collected at E8.5. We extracted total RNAs from the embryos with the RNeasy Protect Mini Kit (Qiagen) and verified the integrity of RNAs with a Bioanalyzer (Agilent Technologies). RNA-Seq libraries were prepared from 500 ng of total RNA by Integragen SA using ribosomal RNA depletion with the Ribo-Zero Gold kit (Epicentre, Madison, WI, USA) and the TruSeq Stranded Total RNA Sample preparation kit (Illumina), followed by paired-end sequencing (2 × 75bp) on an Illumina HiSeq2000. The total numbers of paired-end reads for each library are as follows: WT1, 73,493,661 pairs; WT2, 52,488,499 pairs; WT3, 64,138,495 pairs; KO1, 61,632,013 pairs; KO2, 55,357,040 pairs; KO3, 60,179,105 pairs. We performed quality control checks on sequencing reads with FastQC [57] and aligned reads to the mouse mm10 genome with TopHat2 (v2.0.12) [65]. For data visualization, we generated BigWig files of normalized read counts per base with bam2wig.py in the RSeQC package (v2.4) [66] using only reads that map uniquely in the genome. We calculated raw read counts in mouse RefSeq exons from the BAM files with HTseq-count (v0.6.0) [67] and used these counts to identify differentially expressed genes with DESeq2 (v1.4.5) [68]. Genes were called differentially expressed if the false discovery rate-adjusted *P*-value between WT and knockout was lower than 0.05 and the fold change greater than 2. Normalized counts and Fragments Per Kilobase of exon per Million fragments mapped (FPKM) scores were calculated with the ‘counts’ and ‘fpkm’ functions of DESeq2.

### RT-qPCR

RNAs were reverse transcribed with the Maxima first strand cDNA synthesis kit (Thermo Scientific) using a combination of oligo(dT) and random hexamer primers. RT-qPCR was performed with the Fast SYBR Green Master Mix (Life Technologies) on a StepOnePlus realtime PCR system (Life Technologies). We used fast PCR cycling conditions as follows: 95°C for 20 s, 40 cycles (95°C for 20 s, 64°C for 30 s), followed by a dissociation curve. We performed qPCR measurements in triplicate reactions and normalized to the expression of two housekeeping genes (*Rpl13a*, *Actb*). In parallel we systematically amplified no-RT controls to rule out the presence of contaminating genomic DNA. Primer sequences for qPCR are provided in Additional file 6.

### Data access

The RRBS and RNA-Seq data have been deposited at the NCBI Gene Expression Omnibus database [69] under accession number GSE60334.

## Additional files

Additional file 1: Figure S1. Features of RRBS methylomes in mouse embryos. **Figure S2.** Distribution and kinetics of DNA methylation at custom CpG islands. **Figure S3.** Kinetics of *de novo* methylation at CpG islands in embryos. **Figure S4.** Comparison of CpG island methylation with gene expression in embryos and adult tissues. **Figure S5.** Bisulfite cloning and sequencing of promoter pmCGIs in adult liver. **Figure S6.** Expression of *Dnmt* genes in WT and knockout embryos. **Figure S7.** Analysis of DNMT3B-specific targets in mouse embryos. **Figure S8.** Expression of germline, pluripotency and imprinted genes in *Dnmt3b*-*/*- embryos. **Figure S9.** RRBS methylation profiles at imprinted loci throughout development and in *Dnmt3* knockout embryos. **Figure S10.** CpG island methylation on the X chromosome and at genes that escape X-inactivation in female embryos. Additional file 2: Table S1. List of CpG island methylation scores. Additional file 3: Table S2. High confidence list of methylated promoter CpG islands. Additional file 4: Table S3. List of DNMT3B-dependent regions. Additional file 5: Table S4. Differentially expressed genes in *Dnmt3b* knockout E8.5 embryos. Additional file 6: Table S5. List of primer sequences.

## Abbreviations

CGI: CpG island
DMR: differentially methylated region
DNMT: DNA methyltransferase
E: embryonic day
ES: embryonic stem
gDMR: germline differentially methylated region
pmCGI: partially methylated CpG island
RRBS: reduced representation bisulfite sequencing
RT-qPCR: quantitative RT-PCR
sDMR: somatic differentially methylated region
TSS: transcription start site
WT: wild type
