# Supplementary material for: Ontogeny of CpG island methylation and specificity of DNMT3 methyltransferases during embryonic development in the mouse
Source: Genome Biol. 2014 Dec 3;15(12):545. doi: 10.1186/s13059-014-0545-5 (PMC4295324; doi:10.1186/s13059-014-0545-5)
Supplement: Additional file 1: Figure S1. — Features of RRBS methylomes in mouse embryos. Figure S2. Distribution and kinetics of DNA methylation at custom CpG islands. Figure S3. Kinetics of de novo methylation at CpG islands in embryos. Figure S4. Comparison of CpG island methylation with gene expression in embryos and adult tissues. Figure S5. Bisulfite cloning and sequencing of promoter pmCGIs in adult liver. Figure S6. Expression of Dnmt genes in WT and knockout embryos. Figure S7. Analysis of DNMT3B-specific targets in mouse embryos. Figure S8. Expression of germline, pluripotency and imprinted genes in Dnmt3b-/- embryos. Figure S9. RRBS methylation profiles at imprinted loci throughout development and in Dnmt3 knockout embryos. Figure S10. CpG island methylation on the X chromosome and at genes that escape X-inactivation in female embryos. [file 13059_2014_545_MOESM1_ESM.pdf]

A

Sample	Sex	Paired-end reads	Mapping efficiency (%)	Reads with unique hits (%)	Conversion rate (%)	Median sequencing depth	CpGs 1x	CpGs 8x
Blast E3.5	mixed	22 372 815	88.06	70.47	99.81	40x	1 529 350	1 231 701
Blast E4.5	mixed	31 504 456	89.44	70.47	99.86	59x	1 539 772	1 274 728
Epb E5.5	mixed	30 826 000	88.99	71.31	99.42	57x	1 569 220	1 299 419
Epb E6.5	mixed	30 251 236	90.60	72.80	99.40	62x	1 501 685	1 243 270
Epb E7.5	mixed	30 735 607	93.75	75.90	99.51	69x	1 504 333	1 303 989
E8.5 #1	mixed	90 359 934	89.93	71.16	99.87	162x	1 652 606	1 484 691
E8.5 #2	male	29 248 459	94.36	77.18	99.73	57x	1 518 171	1 289 982
E8.5 #3	female	28,022,970	94.74	75.97	99.84	59x	1 579 261	1 350 159
E8.5 3a-/- #1	male	25 425 189	94.53	76.44	99.76	52x	1 592 272	1 353 151
E8.5 3a-/- #2	female	22 131 450	94.60	76.32	99.86	49x	1 527 352	1 298 445
E8.5 3b-/- #1	female	30 703 665	94.52	75.20	99.94	66x	1 518 520	1 319 926
E8.5 3b-/- #2	female	35 965 036	94.30	75.06	99.94	80x	1 480 417	1 297 828
E10.5 #1	mixed	31 661 954	93.37	76.02	96.62*	69x	1 320 419	1 046 375
E10.5 #2	mixed	34 110 909	93.61	77.43	96.30*	67x	1 376 520	1 074 916
E11.5 limb #1	female	21 789 502	94.89	74.50	99.95	48x	1 474 434	1 218 035
E11.5 limb #2	female	70 673 157	88.88	70.93	99.91	106x	1 678 562	1 393 494
E11.5 limb 3b-/- #1	male	26 476 728	92.85	71.99	99.90	52x	1 511 195	1 262 869
E11.5 limb 3b-/- #2	female	23 500 674	94.19	72.76	99.94	48x	1 513 943	1 255 074
Adult liver	female	28 895 549	86.49	67.58	99.83	46x	1 554 216	1 235 749
Sperm #1	male	19 269 093	91.44	70.11	99.88	42x	1 450 515	1 216 608
Sperm #2	male	33 123 965	91.29	71.56	99.83	63x	1 529 379	1 315 930

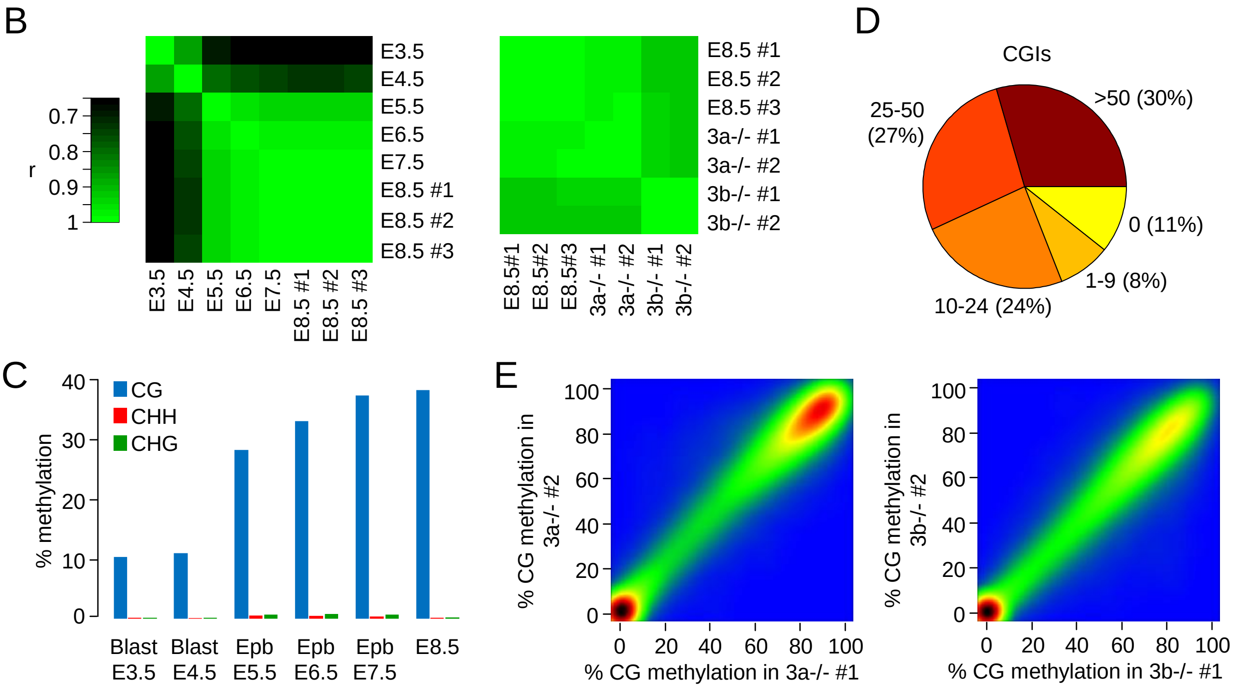

Figure S1. Features of RRBS methylomes in mouse embryos. A) Table summarizing the sequenced RRBS libraries. The last two columns indicate the number of CpGs sequenced at least 1x and 8x obtained from reads that mapped uniquely in the genome. The bisulfite conversion rate was estimated by calculating the C to T conversion at non-CpG sites. The samples marked with an asterisk (*) have a reduced conversion rate because they underwent only one round of bisulfite conversion. Blast=blastocyst; Epb=epiblast. **B)** Correlation matrix showing the Pearson correlation coefficient (r) of methylation at individual CGs between RRBS experiments. **C)** Evolution of the percentage of cytosine methylation at CG, CHH and CHG sites during development (H=A, C or T). **D)** Pie chart representing the coverage of UCSC CpG islands in the Epb E7.5 RRBS dataset. The numbers correspond to how many CpGs were sequenced at least 8x in the CGIs. **E)** Pairwise comparison of CpG methylation levels in 400bp tiles reveals an excellent correlation between biological replicates of RRBS in *Dnmt3a-/-* and *Dnmt3b-/-* embryos.

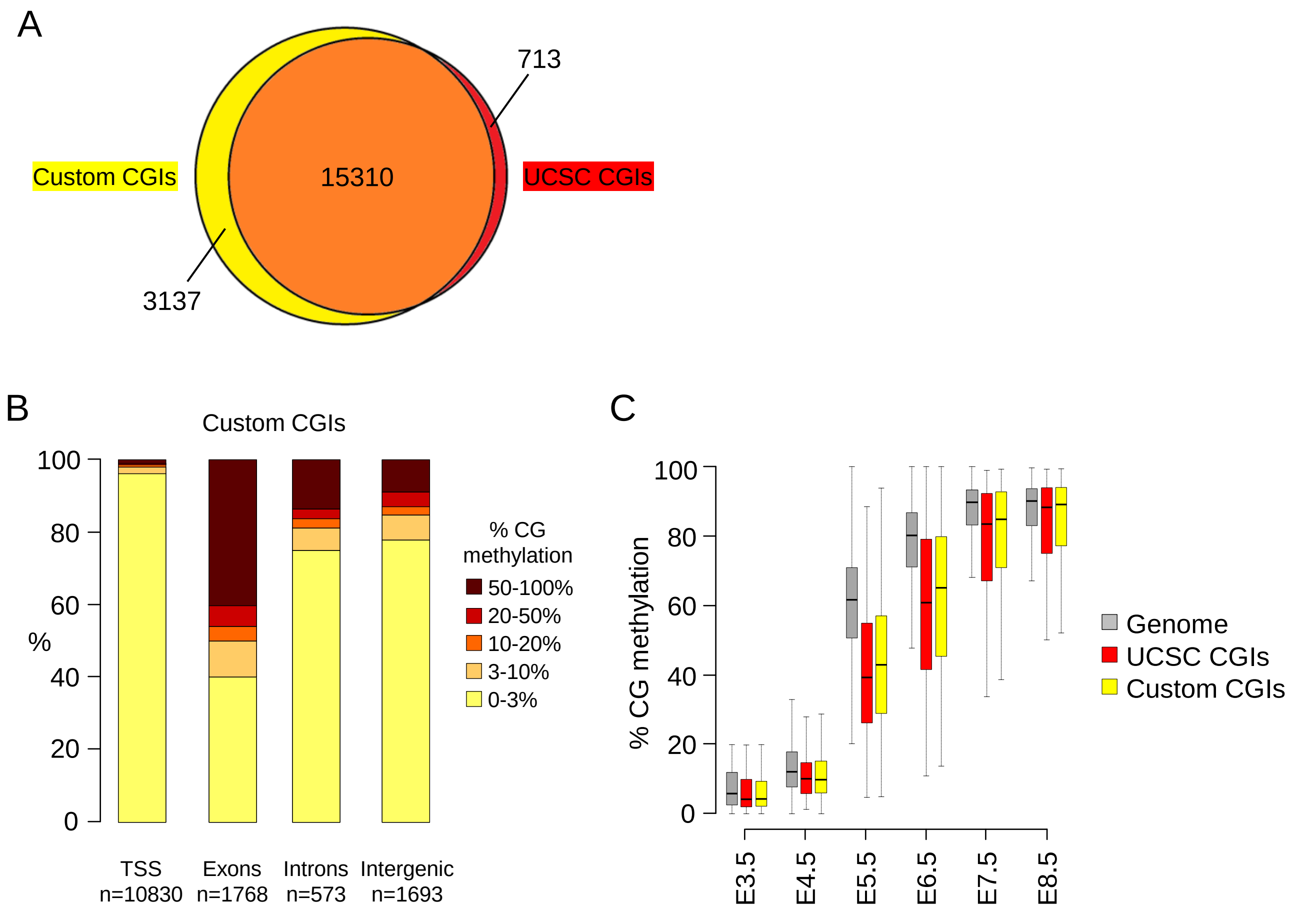

Figure S2. Distribution and kinetics of DNA methylation at custom CpG islands. A) Venn diagram showing the overlap between the UCSC CGIs and the CGIs identified by our custom annotation. **B)** Distribution of E8.5 methylation scores in custom CGIs located in promoters (-1000 to 1000bp from RefSeq TSS), exons, introns and intergenic sequences. X-linked CGIs are excluded from this analysis. **C)** Comparison of the kinetics of *de novo* methylation in genome tiles (400bp), UCSC CGIs and custom CGIs. The graph depicts only *de novo* methylated sequences defined as <20% methylation in E3.5 blastocysts and >50% methylation in E8.5 embryos.

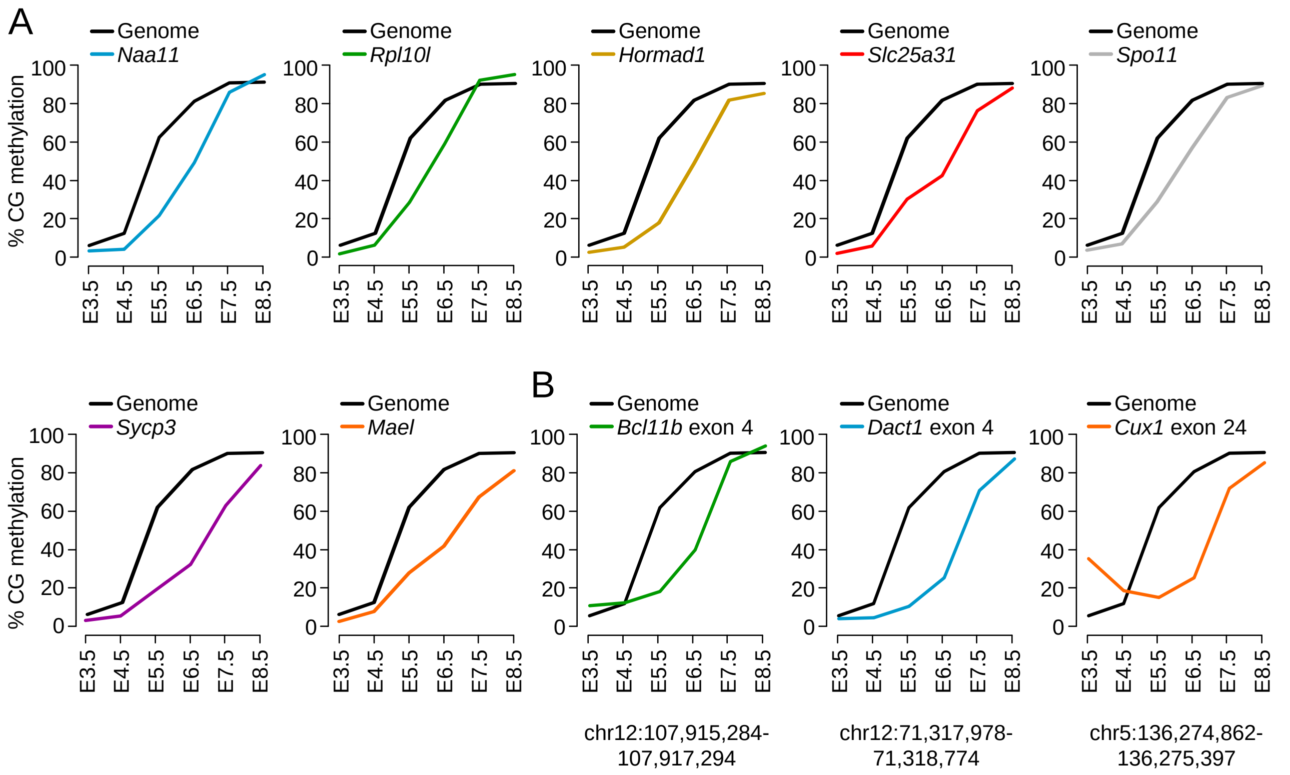

Figure S3. Kinetics of *de novo* methylation at CpG islands in embryos. The graphs show the CpG methylation levels measured at consecutive stages of development in selected CGIs in promoters (**A**) and exons (**B**). The genomic positions of the CGIs in exons are given below the graphs. In each panel, the black line represents the global rate of *de novo* methylation in the genome (calculated in all tiles with <20% methylation in E3.5 blastocysts and >50% methylation in E8.5 embryos).

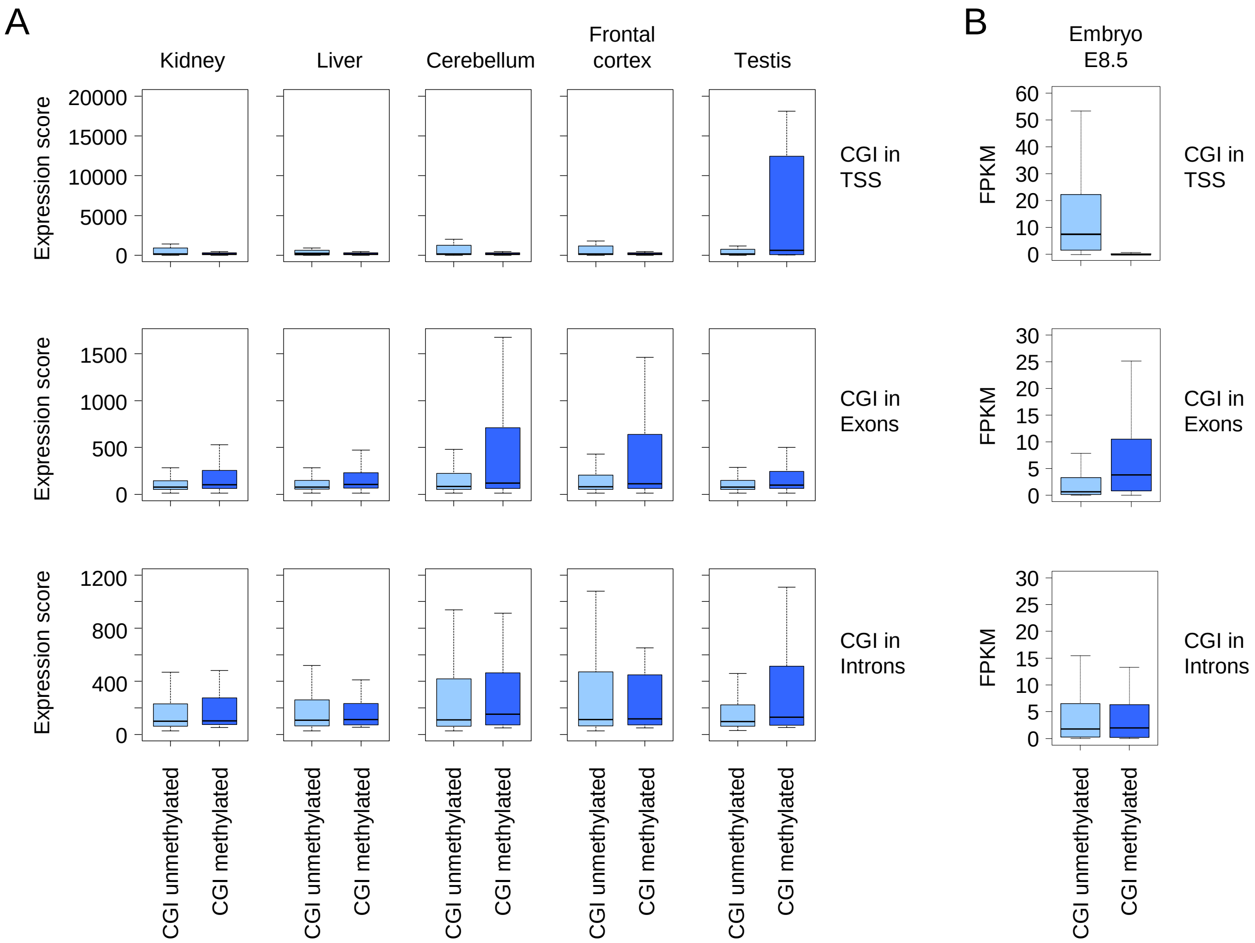

Figure S4. Comparison of CpG island methylation with gene expression in embryos and adult tissues. A) We extracted BioGPS expression scores in adult tissues for all genes with an annotated CGI in the TSS (top), in an exon (middle) or in an intron (bottom). The boxplots show the distribution of expression scores for genes with an unmethylated CGI (<15% methylation in E8.5 embryos, light blue) or a methylated CGI (>50% methylation in E8.5 embryos, dark blue). This shows that genes that gain CGI methylation in the TSS are highly expressed in the testis, whereas genes that gain CGI methylation in an exon show the highest expression in brain tissues. **B)** Similar analysis as in (A) with RNA-Seq FPKM values obtained in wild-type E8.5 embryos.

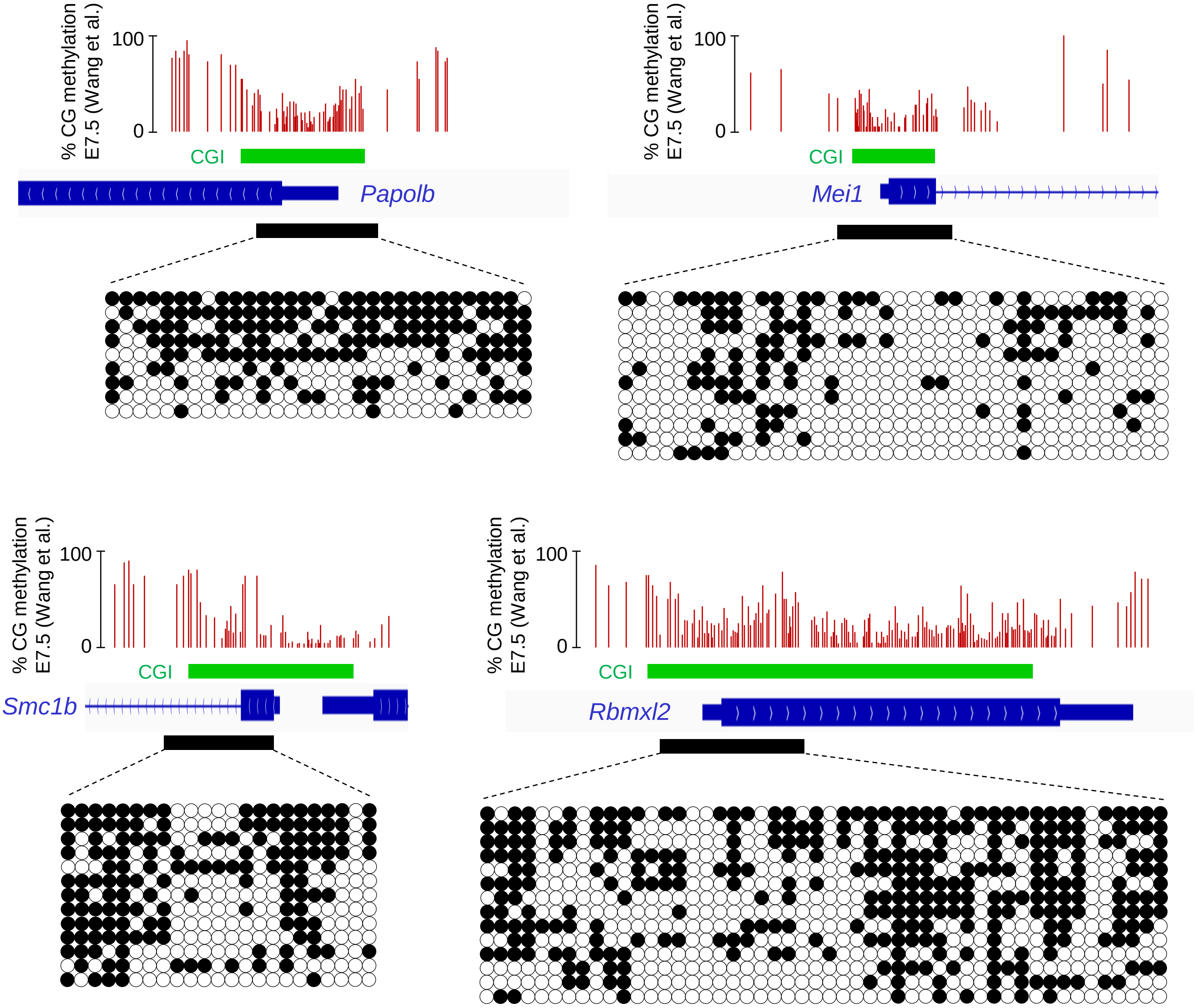

Figure S5. Bisulfite cloning and sequencing of promoter pmCGIs in adult liver. The green bars depict the position of the CGIs and the black bars depict the position of the bisulfite PCR fragments. The circles represent methylated (black) or unmethylated (white) CpG dinucleotides; each horizontal line is one sequenced clone. For comparison we show WGBS data generated in E7.5 embryos (Wang L *et al.* 2014, *Cell* **157**: 979-991).

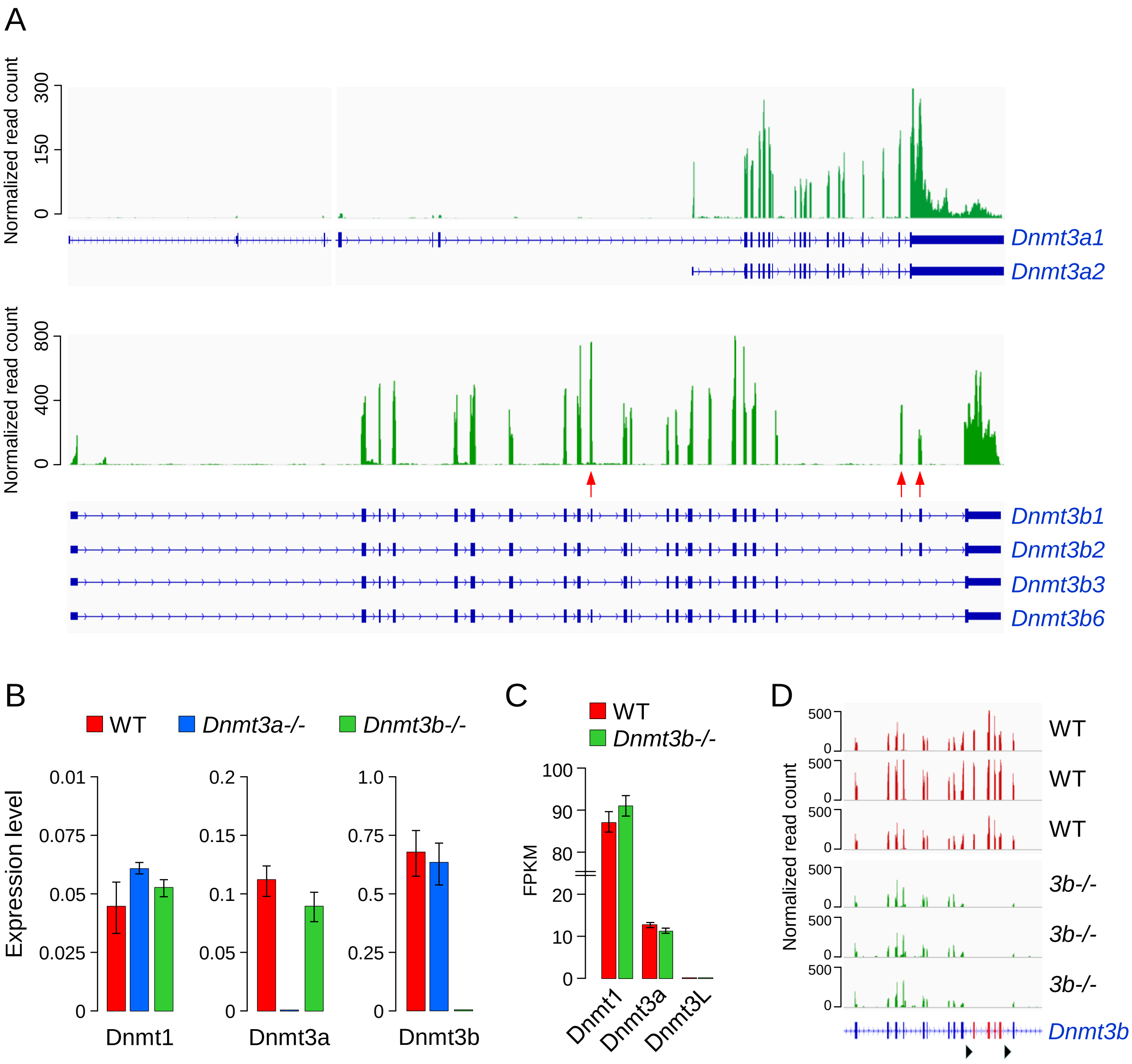

Figure S6. Expression of *Dnmt* genes in WT and knockout embryos. A) RNA-Seq profiles in a WT E8.5 embryo indicate that embryos predominantly express the short *Dnmt3a2* over the long *Dnmt3a1* isoform (top), and the full-length *Dnmt3b1* isoform over the alternative splicing isoforms lacking exon 10, 21 or 22 (marked by red arrows, bottom). **B)** The inactivation of *Dnmt3a* or *Dnmt3b* does not modify the expression of other *Dnmts* in epiblasts. The bar graphs show the expression of *Dnmts* measured by RT-qPCR in WT, *Dnmt3a-/-* and *Dnmt3b-/-* E6.5 epiblasts relative to the expression of the housekeeping gene *Actb*. The error bars represent mean deviations from independent embryos (n=3 for WT, n=2 for KO embryos). For *Dnmt3a* and *Dnmt3b* we used PCR primers that amplify within the Cre-deleted catalytic exons. **C)** Barplot of RNA-Seq FPKM values for *Dnmts* in WT and *Dnmt3b-/-* E8.5 embryos. The error bars represent mean deviations from RNA-Seq in 3 embryos. **D)** Profiles of RNA-Seq read coverage at the *Dnmt3b* locus confirm that the four essential exons flanked by the loxP sites (in red between the black triangles) are absent in *Dnmt3b-/-* embryos.

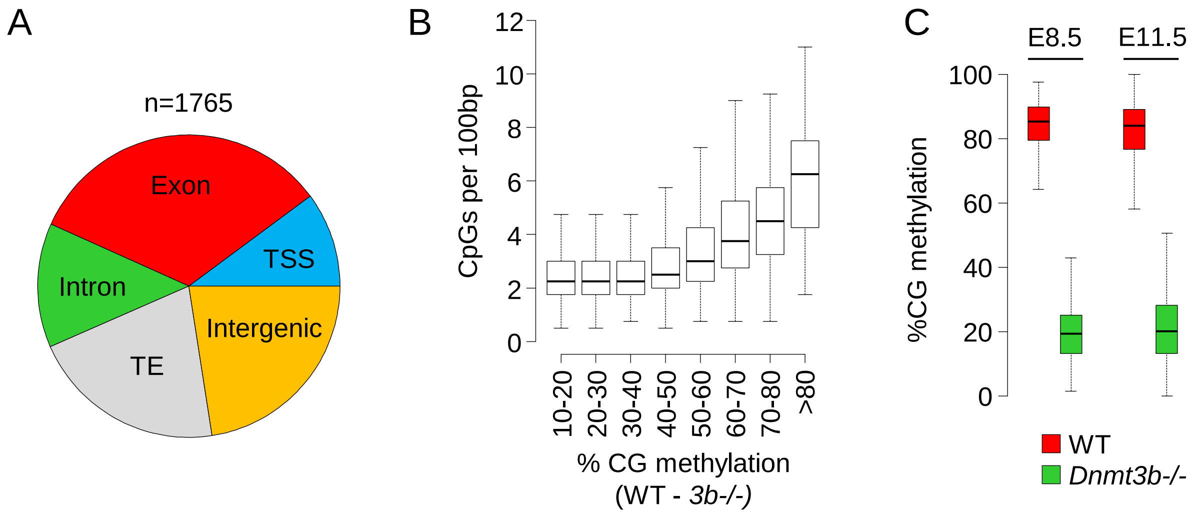

Figure S7. Analysis of DNMT3B-specific targets in mouse embryos. A) Pie chart showing the distribution of DNMT3B-dependent targets (defined as losing more than 60% methylation in *Dnmt3b-/-* compared to WT E8.5 embryos) in TSS, exons, introns, transposable elements (TE) and intergenic regions. **B)** Box plot showing the CpG density (number of CpGs per 100bp, measured in 400bp tiles) as a function of the extent of methylation loss in *Dnmt3b-/-* embryos. The sequences most affected by the inactivation of DNMT3B have an increased CpG density. **C)** Boxplot of CG methylation in E8.5 embryos and E11.5 limbs for all the DNMT3B-dependant targets identified at E8.5.

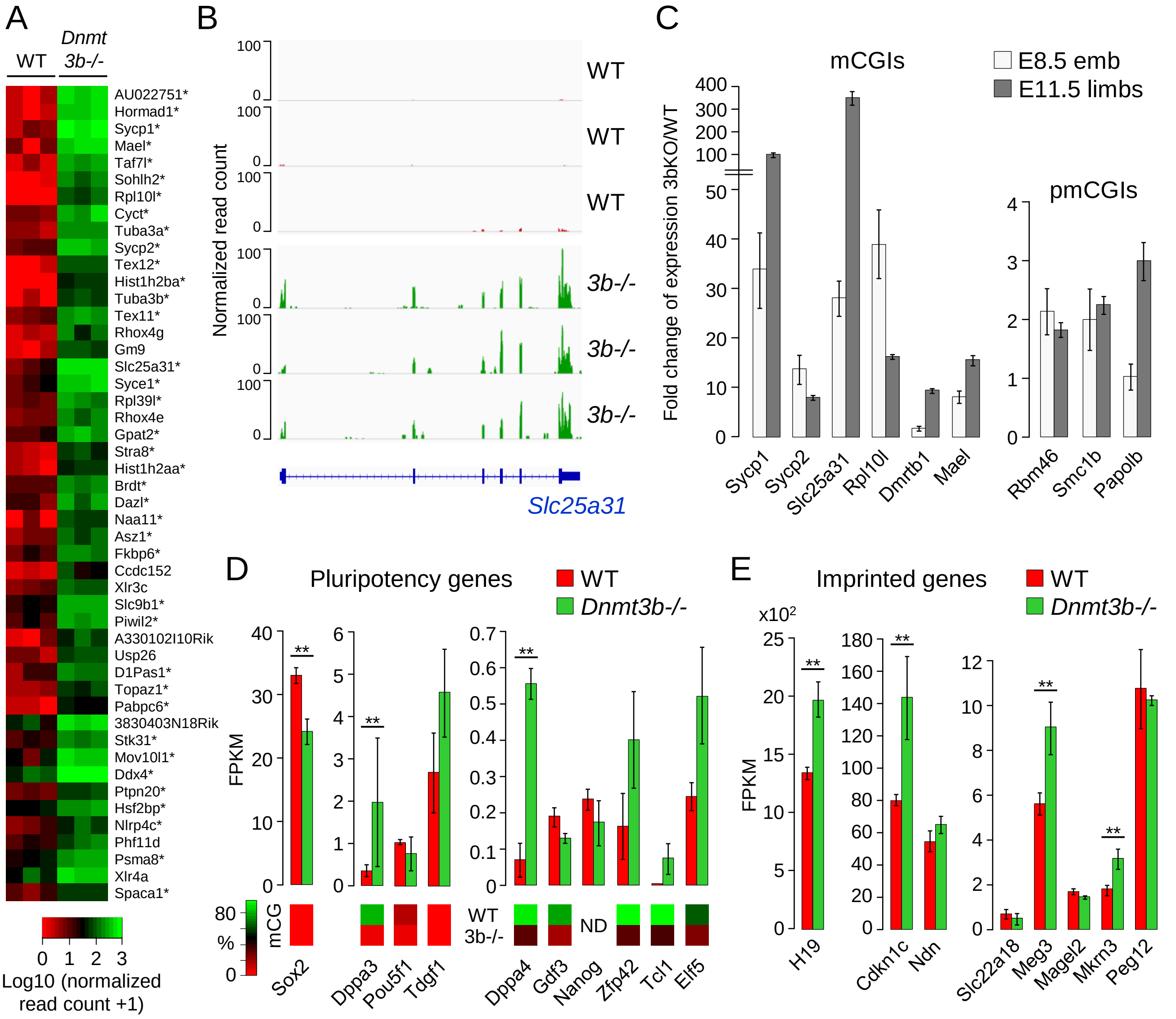

Figure S8. Expression of germline, pluripotency and imprinted genes in *Dnmt3b-/-* embryos. A) Heatmap of DESeq2 normalized read counts for the 48 genes upregulated more than 5 fold in *Dnmt3b-/-* versus WT E8.5 embryos. The asterisks (*) indicate germline-specific genes. **B)** RNA-Seq read coverage at the germline-specific gene *Slc25a31* in WT and *Dnmt3b-/-* embryos. **C)** RT-qPCR analysis of the changes of expression of germline genes in *Dnmt3b-/-* embryos. We measured the expression of germline genes containing a methylated (mCGI) or partially methylated (pmCGI) CGI promoter in E8.5 total embryos and E11.5 limbs. The bar graphs show the fold change of expression in three *Dnmt3b-/-* compared to three WT samples after normalization to the expression of two housekeeping genes (*Actb* and *Rpl13a*). **D)** Barplot of RNA-Seq FPKM values for pluripotency genes in WT and *Dnmt3b-/-* E8.5 embryos. The error bars represent mean deviations from RNA-Seq in 3 embryos. The heatmaps below the graph show the percentage of CG methylation measured by RRBS around the TSS (-1000 to +1000bp) in WT and *Dnmt3b-/-* embryos. ND: not determined. (**) p<0.01 (DESeq2 adjusted p-value). **E)** Barplot of RNA-Seq FPKM values in WT and *Dnmt3b-/-* E8.5 embryos for imprinted genes with sDMRs. The error bars represent mean deviations from RNA-Seq in 3 embryos. (**) p<0.01 (DESeq2 adjusted p-value).

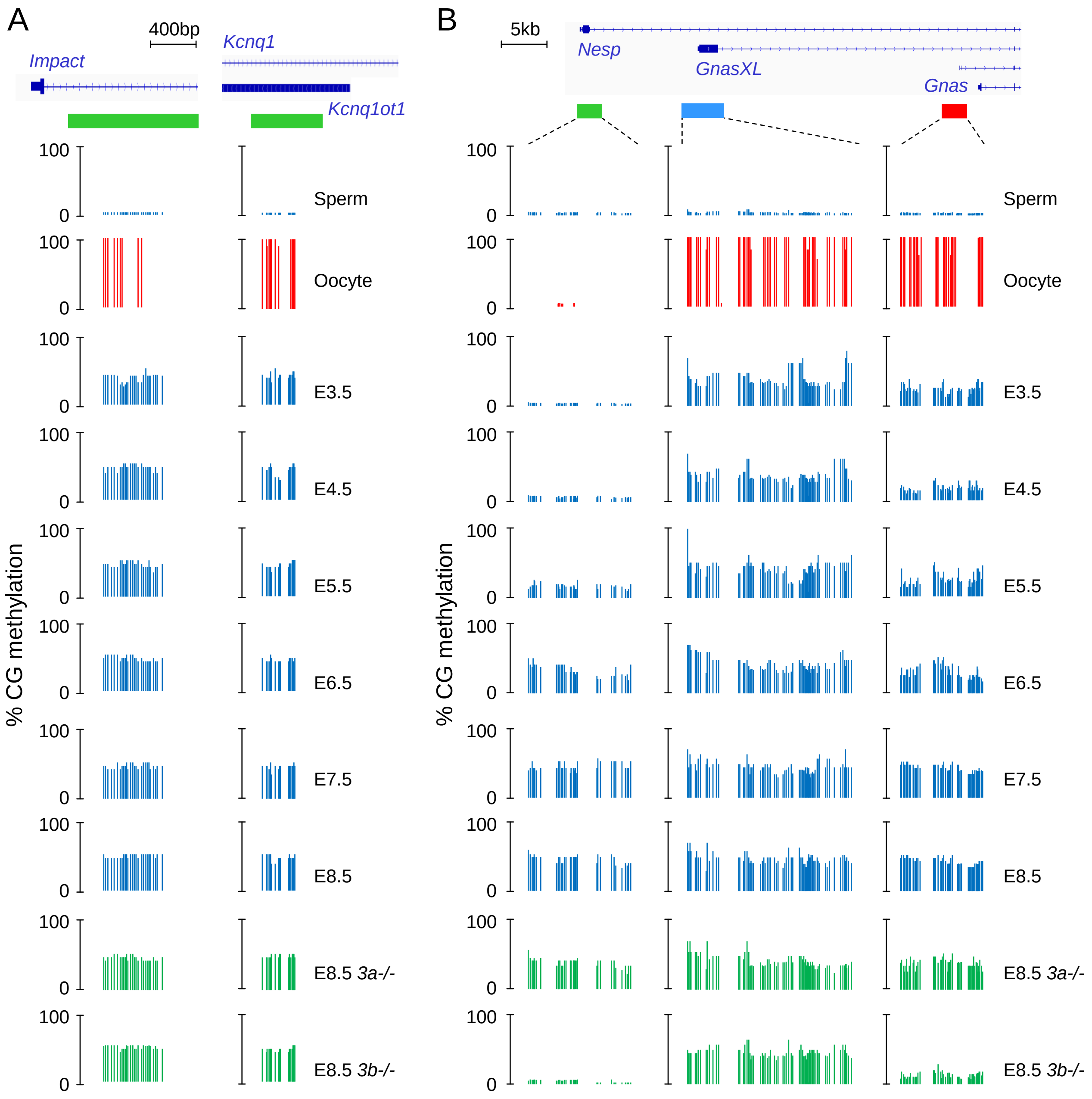

Figure S9. RRBS methylation profiles at imprinted loci throughout development and in *Dnmt3* knockout embryos. A) Single-CpG RRBS profiles at the *Impact* and *Kcnq1* gDMRs that maintain maternal allelic methylation throughout development. The genes are depicted on top of the graphs, and the green bars indicate the position of the gDMRs. **B)** RRBS profiles at the three DMRs of the *Gnas* locus. The structure of the locus is indicated on top of the graphs with the position of the *Nesp* sDMR (green bar), the *GnasXL* gDMR (blue bar) and the *Gnas* Exon1A DMR (red bar).

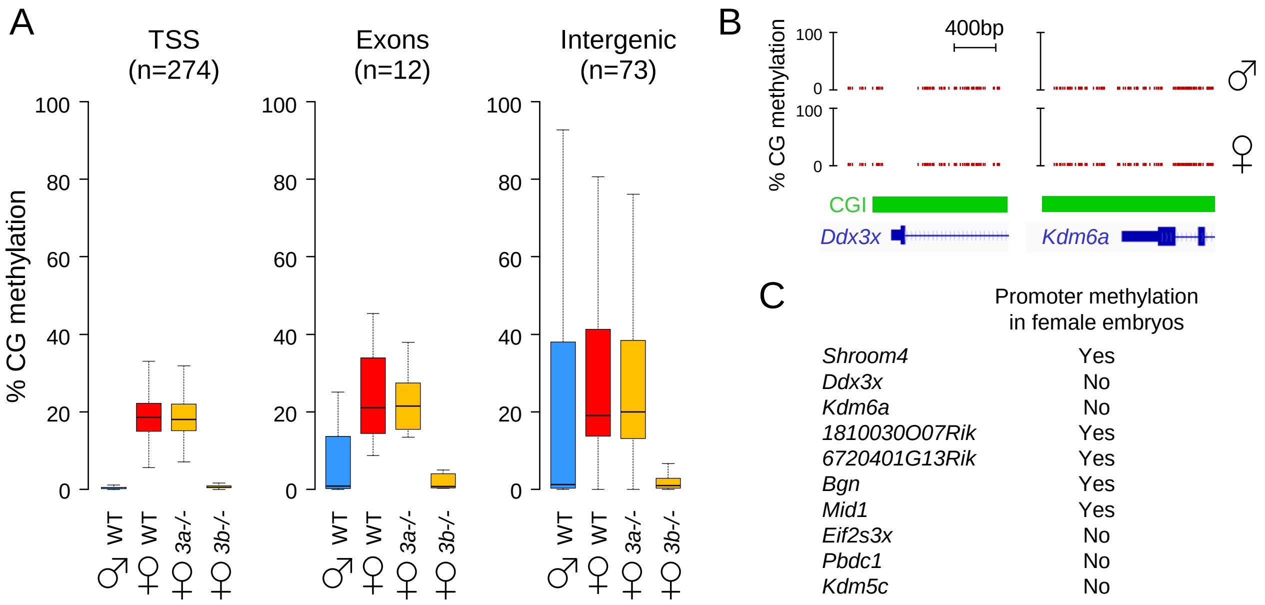

Figure S10. CpG island methylation on the X chromosome and at genes that escape X-inactivation in female embryos. A) Methylation of CGIs in TSS, exons and intergenic sequences on the X chromosome in male and female E8.5 embryos. **B)** RRBS methylation profiles in male and female E8.5 embryos at the CGI promoters of two genes known to escape X-inactivation in mice, which reveals an absence of X-linked methylation in female embryos. The green bars mark the position of CGIs. **C)** Summary of DNA methylation at 10 genes described to escape X-inactivation in mice (Yang F *et al.* 2010, *Genome Res* **20:** 614-622). The table indicates whether our RRBS data revealed a methylation of the gene promoter in female compared to male E8.5 embryos.
